# Biotransformation of Coumarins: Mechanisms and Pharmaceutical Potential

**DOI:** 10.3390/molecules31152584

**Published:** 2026-07-24

**Authors:** Mutiara Saragih, Ewa Szczepańska, Teresa Olejniczak

**Affiliations:** Department of Food Chemistry and Biocatalysis, Faculty of Biotechnology and Food Science, Wrocław University of Environmental and Life Sciences, 50-375 Wrocław, Poland; ewa.szczepanska@upwr.edu.pl

**Keywords:** coumarins, biotransformation, pharmaceutical, microbial transformation, derivatives

## Abstract

Coumarins are compounds that are naturally found in various plants and are known for their potential pharmacological characteristics, such as anti-inflammatory, antibacterial, antiparasitic, and anticoagulant activities. Despite the potential pharmacological properties, structural modification of coumarins is important to enhance the biological activity, solubility, bioavailability, and various therapeutic characteristics. Microbial transformation is a promising method to modify coumarins since this method offers regioselective and stereospecific transformation, which is challenging to obtain through chemical synthesis. Various microorganisms, including bacteria and fungi, play an essential role in the microbial transformation of coumarins. These microorganisms employ enzymatic mechanisms involving various enzymes to catalyze several reactions, such as hydroxylation, oxidation, reduction, and demethylation. Among these microbial transformation processes, the demethylation process is a significant mechanism for altering the bioactivity of coumarins by converting methoxy groups into hydroxyl groups. Despite all the advantages, microbial transformation also has limitations such as low substrate specificity, contamination during inoculation, variable enzymatic activity, and challenges to scale up the production of bioactive coumarins. These challenges can be addressed through the optimization of the bioprocess to enhance the efficiency of microbial coumarin metabolism. This review provides a comprehensive overview of the microbial transformation of coumarins, highlighting the role of various microorganisms, enzymatic mechanisms, and the transformation processes.

## 1. Introduction

### 1.1. Natural Coumarins

**Figure 1:**
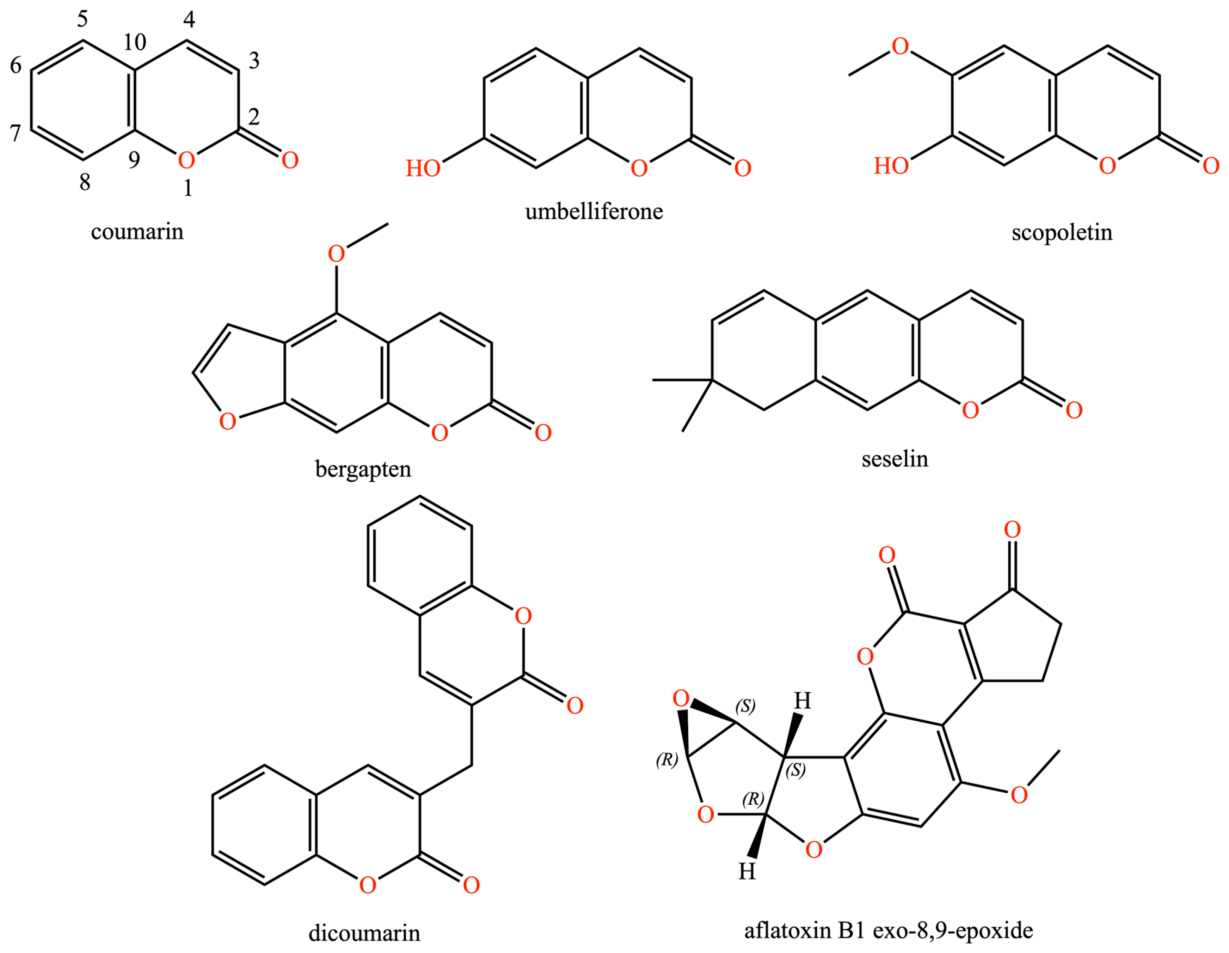
Chemical structures of natural coumarins.

Natural products from plants, animals, and microorganisms have long served as a foundation of drug discovery due to their rich structural diversity, evolutionary optimization, and broad spectrum of bioactivities. Among these natural compounds, coumarins have emerged as one of the most extensively investigated scaffolds, providing both direct pharmacological leads and privileged cores for semisynthetic optimization [1,2,3,4,5]. Chemically, coumarins comprise a benzopyran-2-one system formed by the fusion of a benzene ring with an α-pyrone (Figure 1). The parent compound, coumarin, was first isolated in 1820 from the “tonka bean” (*Dipteryx odorata*; Fabaceae), and its characteristic vanilla-like, freshly mown hay aroma helped drive early applications in perfumery before its potential as a pharmacophore became widely appreciated. In nature, coumarins occur predominantly in higher plants (e.g., tonka bean, cassia, woodruff, lavender oil, peppermint, carrot, green tea, and celery), but they are also produced by bacteria and fungi; notable microbial aminocoumarins include novobiocin and coumermycin from *Streptomyces* species, while difuranocoumarin-type mycotoxins such as aflatoxins are biosynthesized by *Aspergillus* species [6,7,8,9,10,11]. In plants, several coumarins function as phytoalexins, accumulating in response to pathogen attack and contributing to chemical defense [2,9].

From a biosynthetic perspective, the core coumarin framework arises via the shikimate pathway through phenylpropanoid intermediates. Typically, L-phenylalanine is deaminated to trans-cinnamic acid and then converted to *p*-coumaric, caffeic, and ferulic acids; subsequent ortho-hydroxylation and lactonization steps, often mediated by cytochrome P450 monooxygenases and related hydroxylases, yield umbelliferone, scopoletin, and other simple coumarins [2,4,12,13]. This enzymatic concept has been validated in plants as well as endophytic and saprophytic fungi, emphasizing the evolutionary convergence of coumarin assembly and revealing new opportunities for pathway engineering and biotechnological production [4,12,13]. In this context, recent advances in genomics-guided pathway discovery, heterologous expression, and biocatalysis have accelerated access to diverse coumarin analogues and enabled more sustainable production routes [2,4,8,12,14].

Structurally, coumarins (Figure 1) are commonly classified as simple coumarins (e.g., umbelliferone, scopoletin), pyrone-substituted coumarins (e.g., dicoumarol-type 4-hydroxycoumarins), linearly or angularly fused furanocoumarins (e.g., bergapten), and pyranocoumarins (e.g., seselin), with sub-grouping according to ring fusion position and substitution patterns [3,4,5,15]. Substitution at positions 6, 7, and 8, O-alkylation or O-acylation, and annulation with furan or pyran rings modulate physicochemical and biological profiles, enabling fine-tuning of lipophilicity, redox behavior, photo-reactivity, and target engagement [2,3,4]. In line with these structure and function relationships, coumarin derivatives have permeated multiple domains, including fragrances, dyes, optical brighteners, analytical probes, herbicides, and pharmaceuticals [4,10,11,16]. Notably, synthetic 4-hydroxycoumarins form the structural basis of widely used oral anticoagulants (e.g., warfarin and related agents), illustrating how natural product-inspired scaffolds can be developed into clinically transformative drugs [3,5].

In line with their ecological roles and chemical versatility, coumarins exhibit bioactivities relevant to modern therapeutics. Recent studies into natural and semi-synthetic derivatives have revealed their bioactivities, from neuroprotection to antimicrobial and anticancer effects [1,2,3,5,9,17]. These properties are influenced by the molecules’ heteroannulated frameworks and substitution motifs. For example, the integration of electron-donating groups and extended conjugation optimizes redox-modulating and radical-scavenging pathways. Furthermore, the presence of fused furan or pyran rings facilitates DNA intercalation and phototoxicity, which is a crucial mechanism for cancer therapy and photochemotherapy [2,3,4,5]. In parallel, medicinal chemistry has successfully exploited the coumarin core for design, including aromatase inhibitors and enzyme-directed electrophiles, supported by tractable synthetic routes and late-stage diversification [3,5,12]. Concurrently, safety considerations remain important. Certain coumarins display hepatotoxicity at high dietary exposure, and difuranocoumarin mycotoxins (e.g., aflatoxins) pose significant risks to human and animal health, thereby necessitating rigorous exposure control and regulatory oversight [9,15,18].

Their pharmacological and toxicological relevance, coumarins are valued for favorable material properties, including notable thermal stability, strong and tunable optical activity, and photophysical behavior that supports roles as laser dyes, fluorescent reporters, and photo-initiators. These characteristics have catalyzed cross-disciplinary applications spanning chemical biology to agrochemical discovery, such as the recent development of coumarin-containing PPO-inhibiting herbicidal leads [10,11,16]. Looking forward, the convergence of pathway elucidation, synthetic biology, and data-driven medicinal chemistry is expected to further expand the accessible chemical space of coumarins and refine the translation of this venerable natural product class into safe and effective therapeutics [2,3,4,12,13].

### 1.2. Literature Search Methods

A comprehensive literature search was conducted through open access databases, including PubMed, Scopus, and Web of Science, to find relevant studies published from 1982 (only a few refer to the original article related to the mechanism) to 2024. The search used combinations of the following keywords: “coumarin, biotransformation, metabolism, microbial transformation, reaction mechanism, enzymatic reaction, pharmaceutical potential, and structure-relationship activity”. The studies were included if the source focused directly on the chemical and biological mechanisms of coumarin biotransformation or modification, the structural relationship of coumarins, and their pharmaceutical potential.

## 2. Biotransformation of Coumarins

In most microbial transformations, the derivative compounds result from the action of multiple pathways. This review discusses these pathways by each individual process:

### 2.1. Lactone Ring Reduction and Downstream Degradation: Microbial Synthesis of Coumarin-Derived Acids

**Figure 2:**
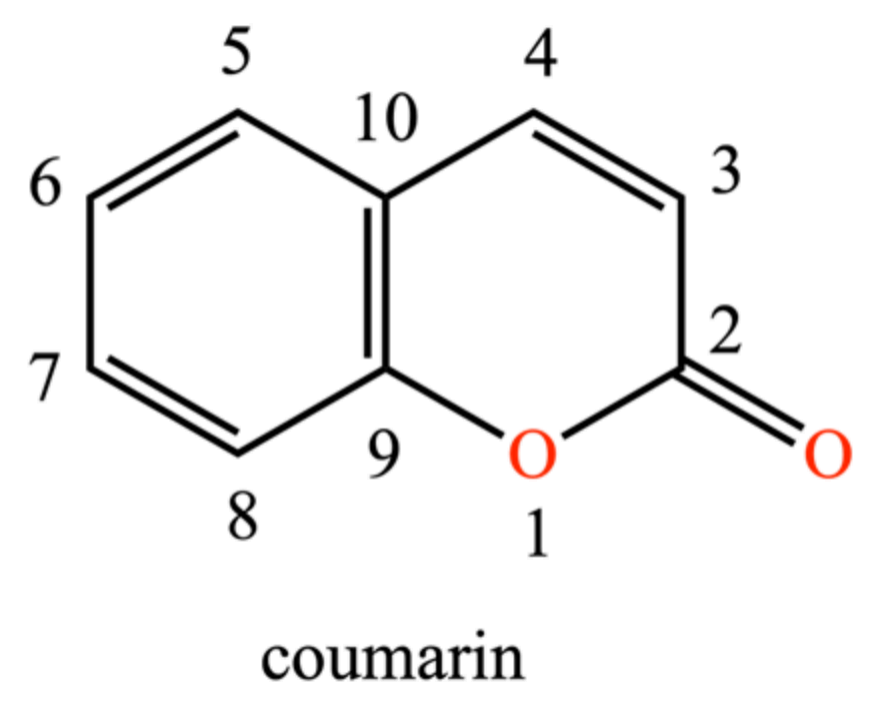
Standard International Union of Pure and Applied Chemistry (IUPAC) atom numbering of the coumarin (2H-chromen-2-one) skeleton with the numbering used throughout this review.

**Figure 3:**
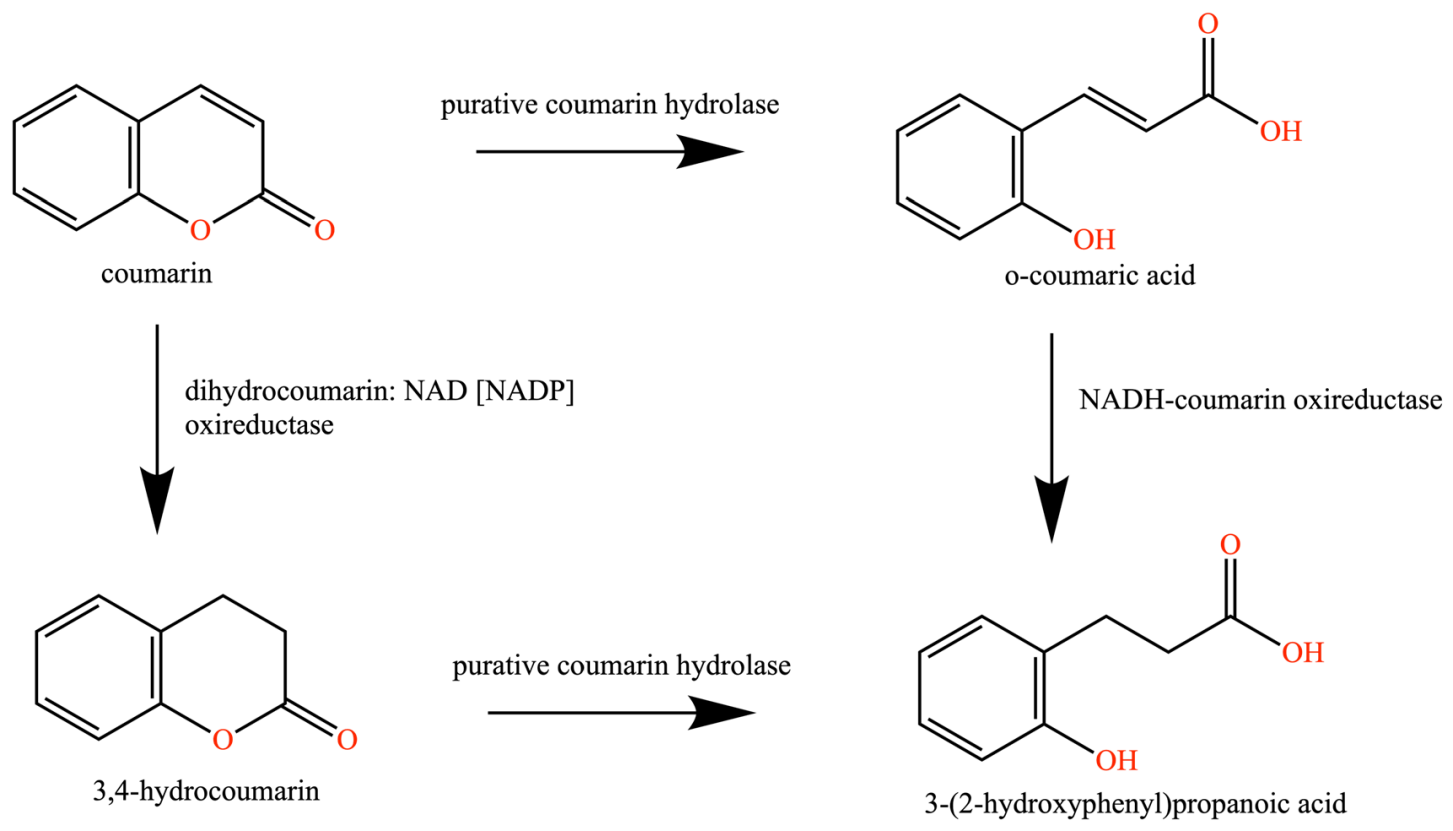
Coumarin metabolic routes in *Arthrobacter* spp., *Pseudomonas* sp. 7HK4, and *Aspergillus* spp.

Bacterial and fungal biotransformation of coumarins results in the formation of corresponding acid derivatives through enzymatic lactone-ring cleavage or reduction. These processes typically involve lactonase- or esterase-mediated hydrolysis to generate β-hydroxycinnamic acids via nucleophilic attack on the C-2 carbonyl and related intermediates that may undergo oxidative degradation or further modification [19]. The reduction reaction via an enzymatic pathway takes place with regio- and stereo-chemical selectivity, which is difficult to achieve in chemical reactions. The enzymatic reactions particularly have an enantioselectivity ability to reduce a carbonyl group to generate a new chiral center [20]. Reduction reactions provide an essential mechanism in coumarin biotransformation, particularly involving the selective hydrogenation or electron transfer of the lactone ring (δ-lactone) or double bond (C-3=C-4) within coumarin compounds (atom positions refer to Figure 2 throughout this section) via a biocatalytic process that leads to the generation of reduced-state products such as dihydrocoumarins [21]. The reduction and oxidation reactions are influenced by pH and oxygen concentration, implying that coumarin compounds contribute to iron metabolism in microbes [22]. A typical example for these coumarins metabolic pathway is transformations using *Arthrobacter* sp., as well as *Pseudomonas* sp. 7HK4, *Aspergillus* sp. and Cunninghamella elegans NRRL 1392, as shown in Figure 3 [23,24].

**Figure 4:**
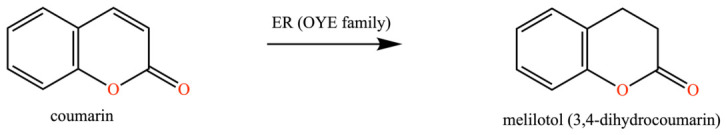
Bicatalytic reduction of α,β-unsaturated lactones with disubstituted C=C bonds using Old Yellow Enzyme (OYE) family.

Trans-cinnamic acid and dihydrocoumarins are formed from coumarins; then these two compounds are converted into 3-(2-hydroxyphenyl) propanoic acid. The reduction reaction occurs in coumarin biotransformation, resulting in the formation of 3,4-dihydrocoumarins. The formation of this derivative occurs exclusively and specifically with coumarin, suggesting narrow substrate specificity of the enzyme involved in this pathway. Finally, cleavage of the coumarin lactone ring leads to trans-cinnamic acid. This pathway occurs through the opening of coumarin’s heterocyclic lactone ring. Furthermore, fungi *Aspergillus* and *C. elegans* also showed activity against substituted coumarins, including umbelliferone, warfarin, and dicumarol. For example, umbelliferone was converted into two derivative compounds: 6,7-dihydroxycoumarin (esculetin) and β-coumaric acid. Esculetin was produced through aromatic hydroxylation of umbelliferone at the C-6 position; moreover, a cleavage reaction of umbelliferone was used to obtain highly polar β-coumaric acid [23,24]. An essential microbial transformation involves the reduction of the C-3=C-4 double bond in the coumarin core, resulting in melilotol, as depicted in Figure 4. This specific reduction is an important step in the formation of dihydrocoumarin derivatives. This compound is a lactone that is essential for fragrance and flavor in industry. Melilotol has a pleasant, sweet, herbaceous odor reminiscent of coumarins and coconut. Given that coumarins are restricted as a food additive due to toxicity concerns, melilotol has attracted attention as a potential alternative flavoring compound. Various biocatalytic reduction pathways were examined using yeasts such as Torulaspora delbrueckii, Kluyveromyces marxianus, and the fungus Penicillium camemberti. Among these microorganisms, K. marxianus showed the highest selectivity and resistance to substrate toxicity, tolerating coumarin concentrations up to 1.8 g/L. For the reduction process of the C-3=C-4 double bond in coumarin, the purified enzyme ene-reductase OYE2 from *Saccharomyces cerevisiae* was used as a biocatalyst using NADPH as the hydride donor, with cofactor regeneration provided by a glucose dehydrogenase (GDH) [25]. OYE2 belongs to the Old Yellow Enzyme (OYE) family of flavin-dependent oxidoreductases, a class of ene-reductases capable of catalyzing the asymmetric reduction of activated C=C bonds [25,26]. This enzymatic approach allowed the use of a higher coumarin substrate concentration of up to 3 g/L, resulting in complete conversion within 72 h and yielding sufficient dihydrocoumarin in 90%. By comparison, the whole-cell microbial reduction utilizing *K. marxianus* is experimentally simpler, but the isolated yield does not exceed 60% [25].

Moreover, reduction is not the only reaction of the C-3=C-4 in the coumarin lactone ring. In mammalian systems, the same group/bond is a substrate for cytochrome P450 (CYP450)-mediated epoxidation rather than reduction, resulting in the reactive coumarin 3,4-epoxide (CE) intermediate. This mechanism has been determined in rodent and human liver microsomes, where CYP1A and CYP2E1 isoforms predominate in hepatic CE formation, and CYP2F2 mediates the epoxidation in lung tissue, with the epoxide altering to o-hydroxyphenylacetic acid as a major metabolite product. This epoxidation pathway is distinct from and competes with the aromatic 7-hydroxylation mechanism, which is catalyzed by CYP2A6 and 3-hydroxylation pathway, which is catalyzed by CYP3A or CYP1A. In addition, this pathway underlies coumarin’s species-dependent hepatotoxicity rather than direct hydroxylation. Despite the demonstrated capacity of filamentous fungi to catalyze analogous epoxidation on the side chain of coumarin, for instance, the C-6 and C-7 olefinic epoxidation examined in *Mucor polymorphosporus,* which reacts on the terpenoid side chain of auraptene, epoxidation of the pyrone ring of the double bond has not been reported as a common microbial transformation mechanism, in contrast to the reduction and hydroxylation mechanisms. This finding showed that microbial systems capable of core-ring epoxidation could provide a biocatalytic pathway to access coumarin epoxide without depending on animal-derived microsomal preparations and would be the target for the investigation of the mechanistic and toxicological importance of this mechanism in mammalian metabolism [27,28].

**Figure 5:**
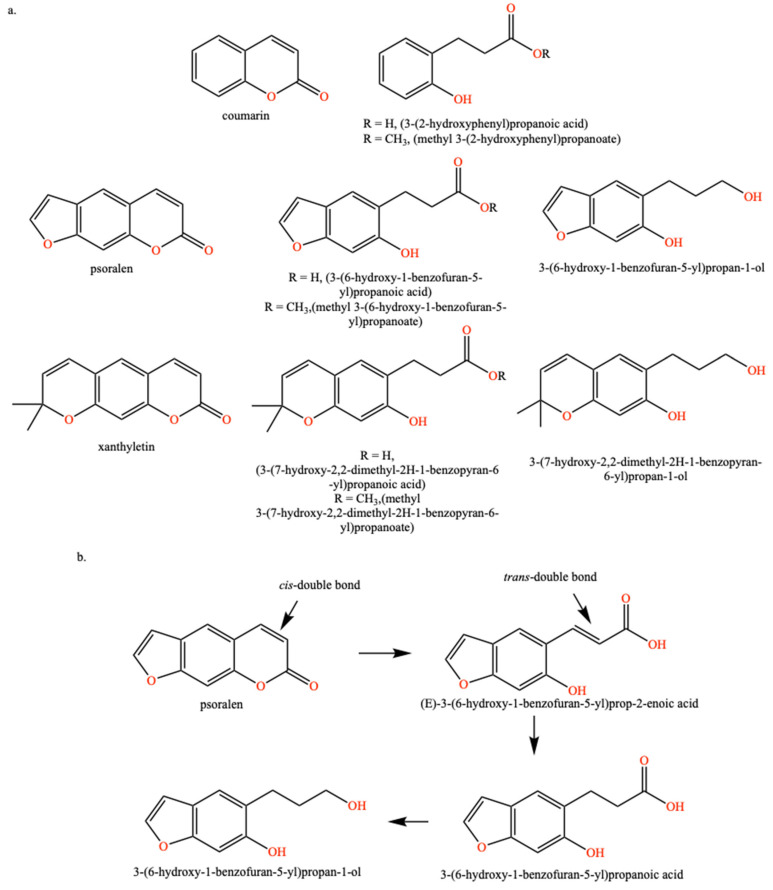
(**a**) Chemical structures of coumarin, psoralen, and xanthyletin derivatives, (**b**) microbial transformation process of psoralen.

Furthermore, the filamentous fungus *Glomerella cingulata* NBRC 5952 was identified as an important biocatalyst for the microbiological conversion of coumarin, psoralen, and xanthyletin through the reduction of the α,β-unsaturated δ-lactone ring of various coumarin analogs as the main mechanism. This fungus metabolized the coumarin compound, forming reduced hydrocoumaric acid or its methyl ester. Similarly, 3-(6-hydroxy-1-benzofuran-5-yl)propanoic acid and methyl 3-(6-hydroxy-1-benzofuran-5-yl)propanoate were produced from psoralen, and 3-(7-hydroxy-2,2-dimethyl-2H-1-benzopyran-6-yl)propanoic acid and methyl 3-(7-hydroxy-2,2-dimethyl-2H-1-benzopyran-6-yl)propanoate were formed from xanthyletin as illustrated in Figure 5. This study demonstrated that the reduction process carried out by the fungus proceeded through the opening of the coumarin lactone ring, followed by isomerization and hydrogenation of the double bond at positions 3,4. This microbial transformation process catalyzed by G. cingulata occurs in three sequential reaction steps: the first step involves hydrolysis of the six-membered cyclic lactone ring, a process combined with isomerization of the double bond; the second step involves reduction of the double bond; and the third step is the reduction of the acid to the corresponding alcohol. Additionally, esterification products of the acids were also observed [29]. It is known that all coumarins are extremely stable in their lactone form, and reduction of the 3,4 double bonds of coumarin lactone rings would be difficult to achieve. Therefore, fungal reduction of the C-3=C-4 bond in coumarins certainly proceeds through lactone ring opening, followed by isomerization (to *trans* stereochemistry). Regarding the evaluation of biological activity, the methyl ester derivatives of metabolites exhibited stronger β-secretase inhibitory activity.

Peucedanin and oreoselone are classified as furanocoumarins, which are produced by Apiaceae vegetables, citrus fruits, and some herbal medicines. These furanocoumarins commonly act as photosensitizing agents, giving them favorable pharmacological properties for the treatment of skin conditions [6,30,31,32]. Biotransformation of oreoselone was carried out by *Aspergillus* sp. PTCC 5266. Oreoselone contains one α,β-unsaturated δ-lactone ring moiety (7H-furo[3,2-g] chromen-7-one), which is the main site where fungal transformation occurs through a hydrolytic ring-opening reaction. The products of this microbial transformation are reduced and lactone-ring-opened methyl 3-(2,3-dihydro-6-hydroxy-2-isopropyl-3-oxobenzofuran-5-yl) propanoate. The lactone ring hydrolysis reaction is carried out with the participation of an esterase/hydrolase. This enzyme performs a nucleophilic attack using a water molecule or a hydroxyl group on the lactone’s C-2 carbonyl group. In this reaction, the methyl ester present in the final product indicates that the carboxylic acid was methylated by a methyltransferase [6,33,34].

**Figure 6:**
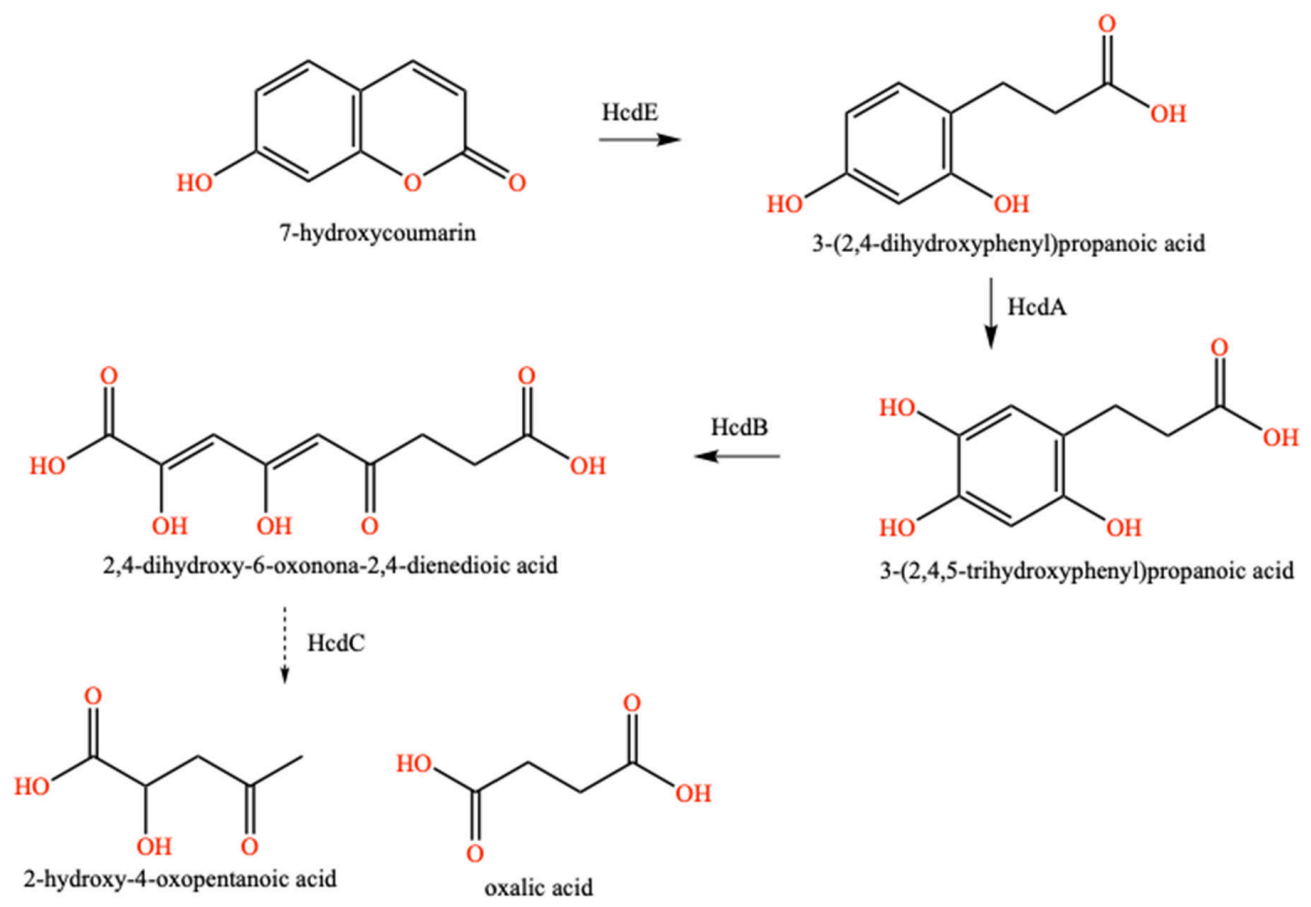
Microbial transformation of 7-hydroxycoumarin by the HcdABC operon in *Pseudomonas mandelii* 7HK4.

Similarly, the bacterial *Pseudomonas mandelii* 7HK4 has been identified as a soil microorganism capable of degrading 7-hydroxycoumarin through a specific pathway that leads to acid formation. The biochemical determination revealed that this strain uses a cluster of genes, *hcdABC* (hydroxycoumarin-degrading operon), which encodes three essential enzymes: flavin-binding hydrolase (HcdA), extradiol dioxygenase (HcdB), and putative hydroxymuconic semialdehyde hydrolase (HcdC). These enzymes collectively catalyze the degradation of 3-(2,4-dihydroxyphenyl)-propionic acid, an intermediate compound produced from 7-hydroxycoumarin. This biotransformation process by this bacterial is catalyzed by the ipso-hydroxylase HcdA, which alters 3-(2,4-dihydroxyphenyl)-propionic acid into 3-(2,3,5-trihydrocyphenyl)-propionic acid via ipso-hydroxylation followed by a 1,2-C,C shift, also known as the NIH shift, as shown in Figure 6. The subsequent reaction, catalyzed by HcdB, which mediates oxidative meta-cleavage of the aromatic ring, results in the linear compound (2E,4E)-2,4-dihydroxy-6-oxonona-2,4-dienedioic acid. In the final step, the HcdC enzyme hydrolyzes this ring-cleaved intermediate to complete the degradation process toward less complex acid derivatives [23,29,35].

In addition, the biotransformation pathway of 7-hydroxycoumarin in *P. mandelii* 7HK4 gives critical insights into the microbial degradation of natural coumarins in the environment. The determination of HcdE, an alcohol dehydrogenase enzyme that is responsible for the first step of the reduction reaction, shows a novel enzymatic mechanism for the catabolism of α,β-unsaturated lactones. Ultimately, the complete mechanism, from the first step of ring reduction and opening to the final step of aromatic ring cleavage, provides the metabolic versatility of *Pseudomonas* species to degrade environmental xenobiotics and natural organic substances [23].

### 2.2. Hydroxylation of Coumarins: Microbial Hydroxylation Introduces Polar Groups and Enhances Bioactivity

Hydroxylation is one of the essential reactions in microbial transformation. This reaction involves the enzymatic addition of a hydroxyl group (-OH) onto an organic substrate. The insertion of an OH group is critical because this pathway can increase the compound’s polarity and solubility, which are important changes to facilitate the drug’s excretion from the organism or to alter the chemical scaffold to enhance the bioactivity. Microorganisms, such as fungi and bacteria, are essential for this process since their enzyme systems perform hydroxylation in a regioselective and stereospecific manner, which allows for precise conversion of complex compounds. Commonly, CYP450 enzymes are responsible for catalyzing the hydroxylation process in biotransformation. These enzymes are heme-containing enzymes that act as mixed-function oxidases, which require oxygen (O_2_) and reduced co-factors, such as NADPH. The ο-hydroxylation is a direct process of hydroxylation, in which the direct insertion of an OH group onto an existing C-H bond occurs, like the hydroxylation of an aromatic ring (at C-6 or C-7, see Figure 2) or an aliphatic side chain [36]. One of the essential advantages of the microbial transformation process is the ability of microorganisms to hydroxylate substrates at chemically inaccessible positions. This ability is a key mechanism to functionalize of inactive C-H bonds, which is the big challenge in organic synthesis. Conventional organic synthesis methods require reactive oxidizing agents, leading to difficulties in controlling the regioselectivity and stereoselectivity of the reactions. Additionally, the esterification process of certain compounds can also be facilitated by microbial transformation [37,38].

**Figure 7:**
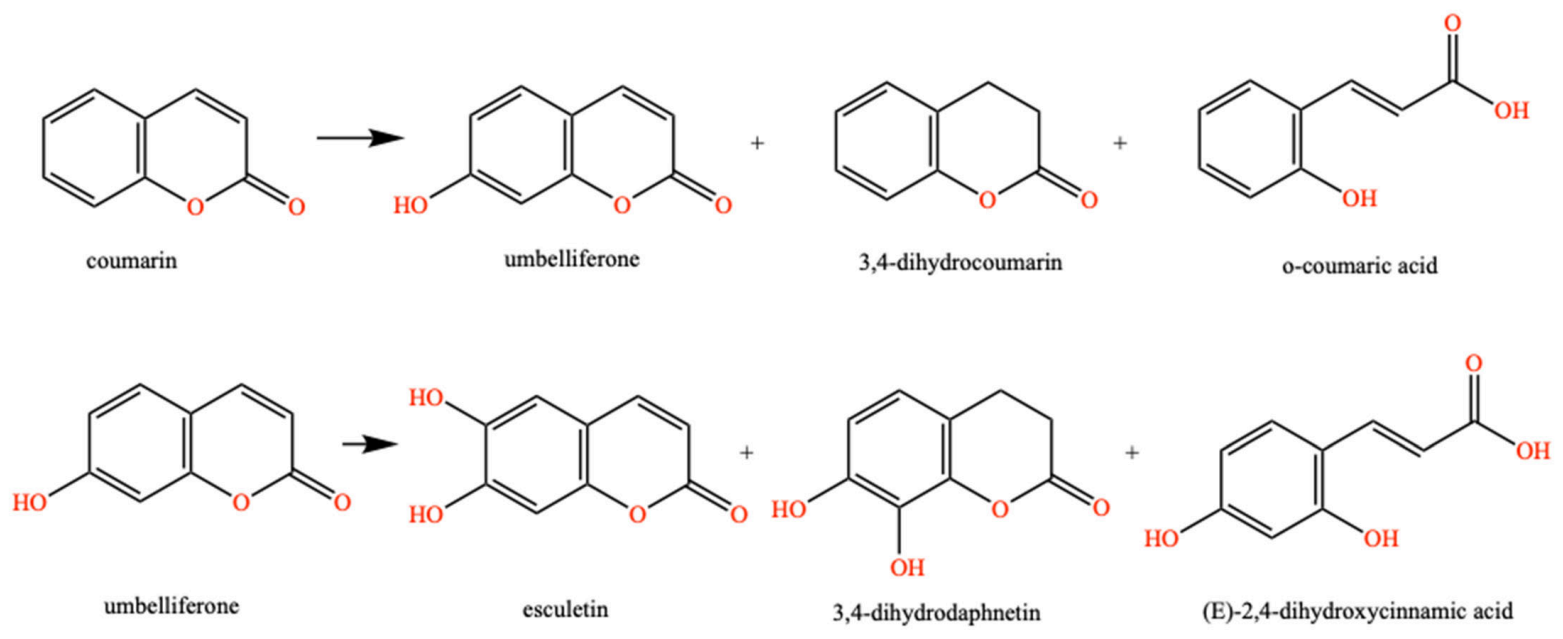
Biotransformation of coumarins by *Cunninghamela elegans*.

Previous research examined the microbial transformation of coumarins using 25 microorganisms, including *Aspergillus*, *Cunninghamella*, *Gymnascella*, *Lindera*, *Penicillium*, *Rhizopus*, *Rhodotorula*, *Fusarium*, *Candida*, and *Saccharomyces genera*. Among these, the fungus *Cunninghamela elegans* NRRL 1392 efficiently metabolized coumarins into umbelliferone, as well as 3,4-dihydrocoumarin and transcinnamic acid. Furthermore, the umbelliferone was also transformed into 6,7-dihydroxycoumarin (aesculetin) and *p*-coumaric acid. The research on the microbial transformation of these coumarins by using *C. elegans* presented that this strain catalyzed the hydroxylation and opening of the lactone ring. In addition, the cytotoxicity of all derivative compounds was tested against a tumor cell line, revealing that the substrates were metabolized into less toxic compounds, except for 6,7-dihydroxycoumarin and 3,4-dihydrocoumarin, as shown in Figure 7 [39].

**Figure 8:**
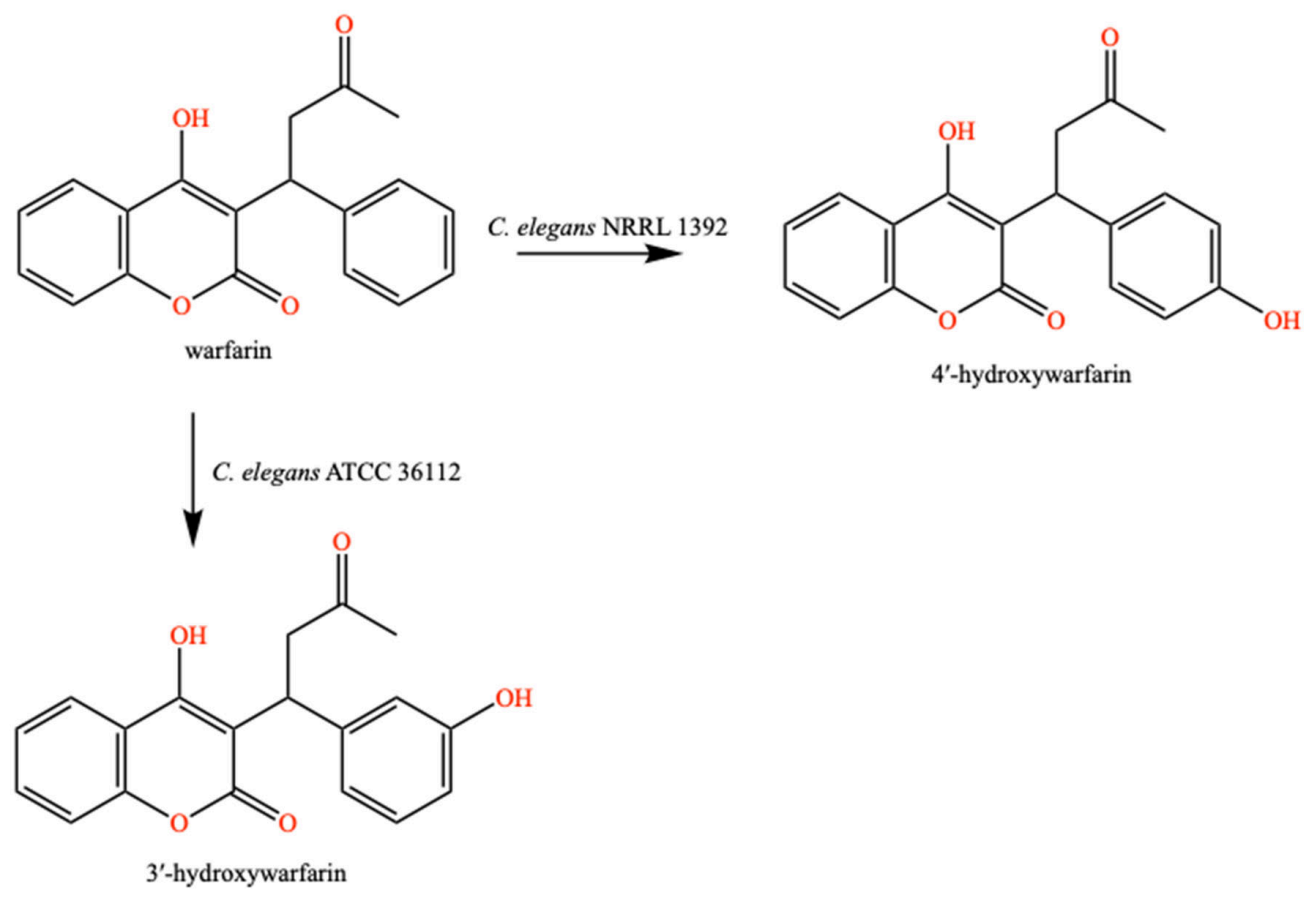
Microbial hydroxylation of coumarin derivatives by *Cunninghamella elegans* strains (NRRL 1392 and ATCC 36112), yielding hydroxy-substituted products.

Moreover, another strain of *Cunninghamela elegans* ATCC 36112 has the potential for stereoselective biotransformation of warfarin. This strain was shown to produce all metabolites of warfarin, including 4′-, 6-, 7-, and 8-hydroxywarfarin, as well as the diastereomeric warfarin alcohols, warfarin diketone, and aliphatic hydroxywarfarin as depicted in Figure 8. Using S-warfarin and R-warfarin as substrates, the microbial transformation of warfarin was demonstrated to be highly stereoselective toward both substrate and product. Moreover, aromatic hydroxylation and ketone reduction showed stereoselectivity toward R-warfarin. In addition, reduction of the ketone of the warfarin enantiomers exhibited product stereoselectivity, with R-warfarin being reduced to its S-alcohol form. In parallel, S-warfarin yielded the corresponding R-alcohol [40]. 

**Figure 9:**
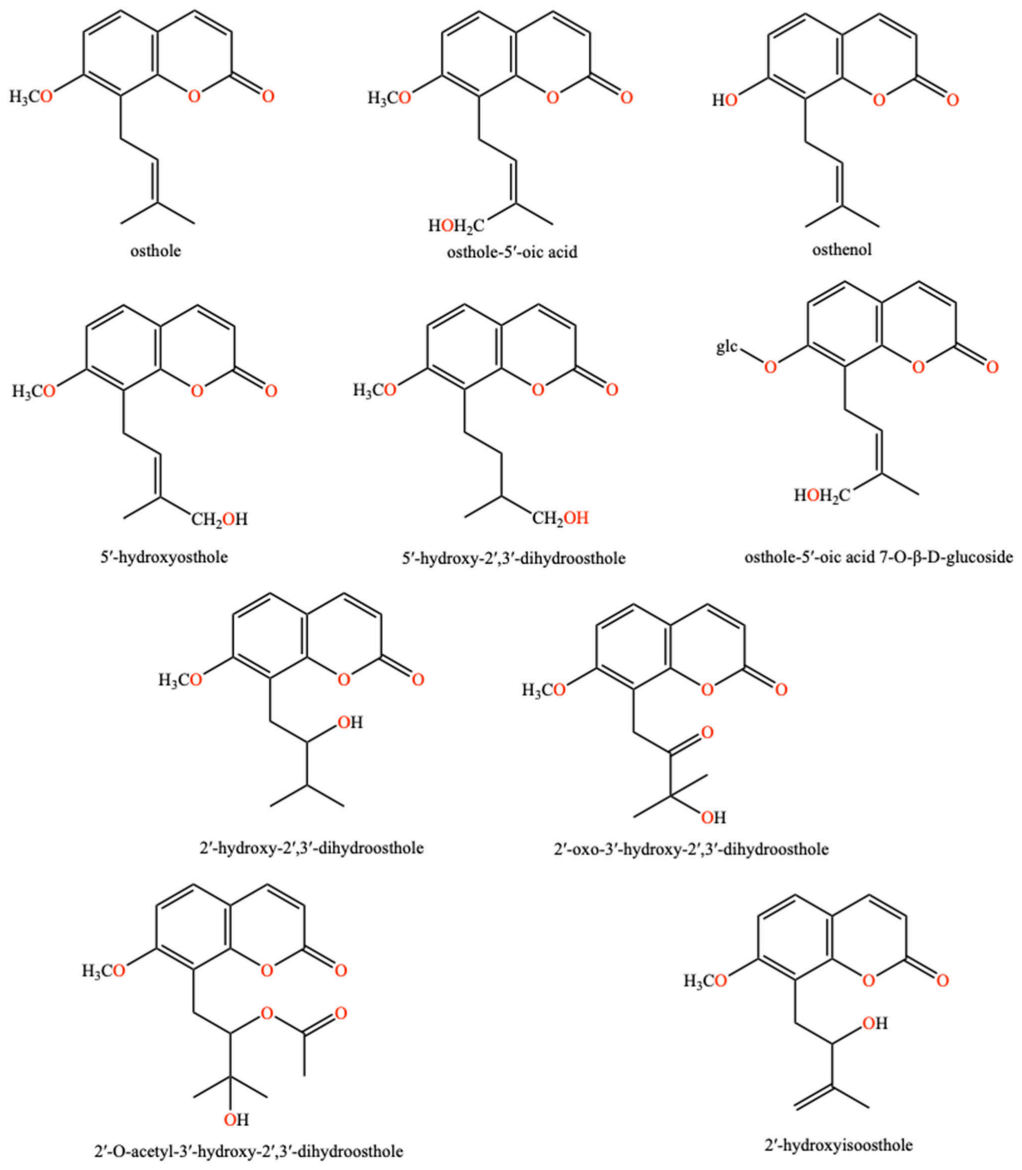
Chemical structure of derivative compounds produced through microbial transformation of osthole using *Mucor spinosus*.

Osthole (7-methoxy-8-2-butenyl)-2H-1-benzopyran-2-one) is a coumarin derivative, which was originally isolated from *Cnidium monnieri* (L.) Cusson. Recently, this coumarin has received a lot of clinical attention since it has promising pharmacological properties on neuroprotection, anti-cancer, anti-inflammatory, and antimicrobial effects [41]. Previous research conducted the microbial transformation of osthole by using *Mucor spinosus* AS 3.3450 and obtained nine derivatives compounds, including osthole-5′-oic acid, osthenol, 5′-hydroxyosthole, 5′-hydroxy-2′,3′-dihydroosthole, osthole-5′-oic acid 7-O-β-D-glucoside, 2′-hydroxy-2′,3′-dihydroosthole, 2′-oxo-3′-hydroxy-2′,3′-dihydroosthole, 2′-O-acetyl-3′-hydroxy-2′,3′-dihydroosthole, and 2′-hydroxyisoosthole as illustrated in Figure 9. The reaction mechanism proceeds through hydroxylation, dehydrogenation, demethylation, and glycosylation, leading to an increase in polarity and water-solubility of derivative compounds. Specifically, the hydroxylation occurred at C-2′, C-3′, C-4′, and C-5′ positions of the isopentene side chain. Demethylation at C-7 was the major metabolic pathway of this microbial transformation to yield osthenol. Subsequently, the glycosylation of osthenol resulted in the glycosylated metabolite, osthenol-7-O-β-d-glucoside. Furthermore, the *Z*-configuration at the double bond of 4-hydroxyl-osthole influences molecular interactions that enhance bioactivity relative to the *E*-configuration in 5-hydroxyl-osthole [42,43].

**Figure 10:**
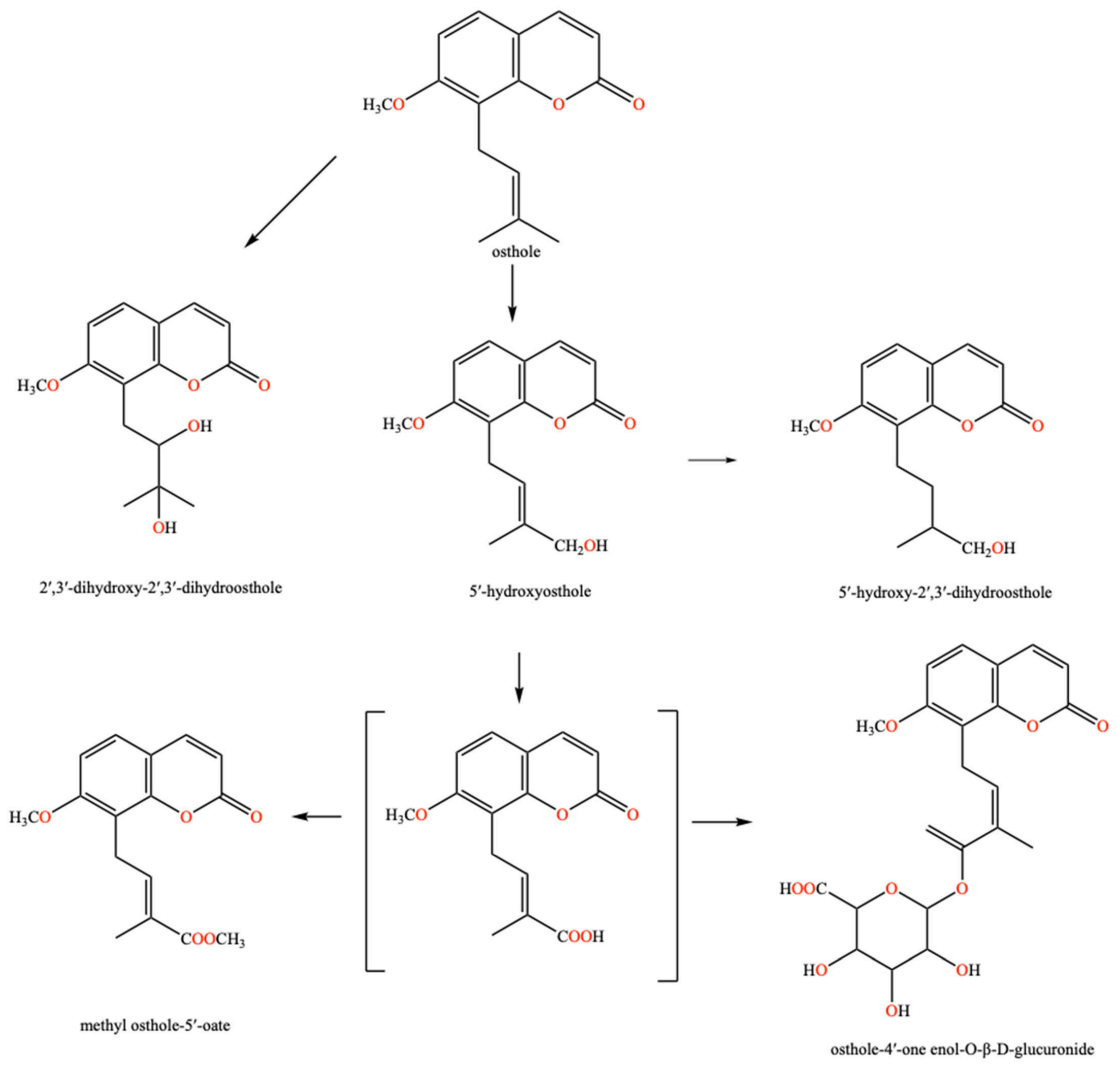
Possible microbial transformation pathway of osthole.

Similarly, osthole compound was carried out through a biotransformation process using *Alternaria longipes* and resulting derivatives such as 2′,3′-dihydroxy-2′,3′-dihydroosthole, 5′-hydroxyosthole, 5′-hydroxy-2′,3′-dihydroosthole, methyl osthole-5′-oate, and osthole-4′-one enol-O-β-D-glucuronide. By obtaining compound Methyl osthole-5′-oate, a methoxycarbonyl group was indicated at the C-4′ position, and the substitution of a methoxyl group occurred at the C-7′ position. In this microbial transformation, the formation of the compound osthole-4′-one enol-O-β-D-glucuronide resulted from the complex glucuronidation pathway, which is a rare occurrence in biotransformation. This glucuronidation reaction involved the conjugation of the glucuronic acid moiety at the C-4′ position with β-configuration. Moreover, the presence of a hydroxyl group was elucidated in compound 5′-Hydroxyosthole at the C-4′ position, proving the hydroxylation process. Additionally, the loss of the carbon double bond was determined, accompanied by the insertion of two hydroxyl groups at C-2′ and C-3′ positions, resulting in the formation of compound 8-(2,3-dihydroxy-3-methylbutyl)-7-methoxy-2H-chromen-2-one via the hydroxylation of the double bond in the isopentenoxy group. The possible microbial transformation pathway of osthole was illustrated in Figure 10 [44,45].

**Figure 11:**
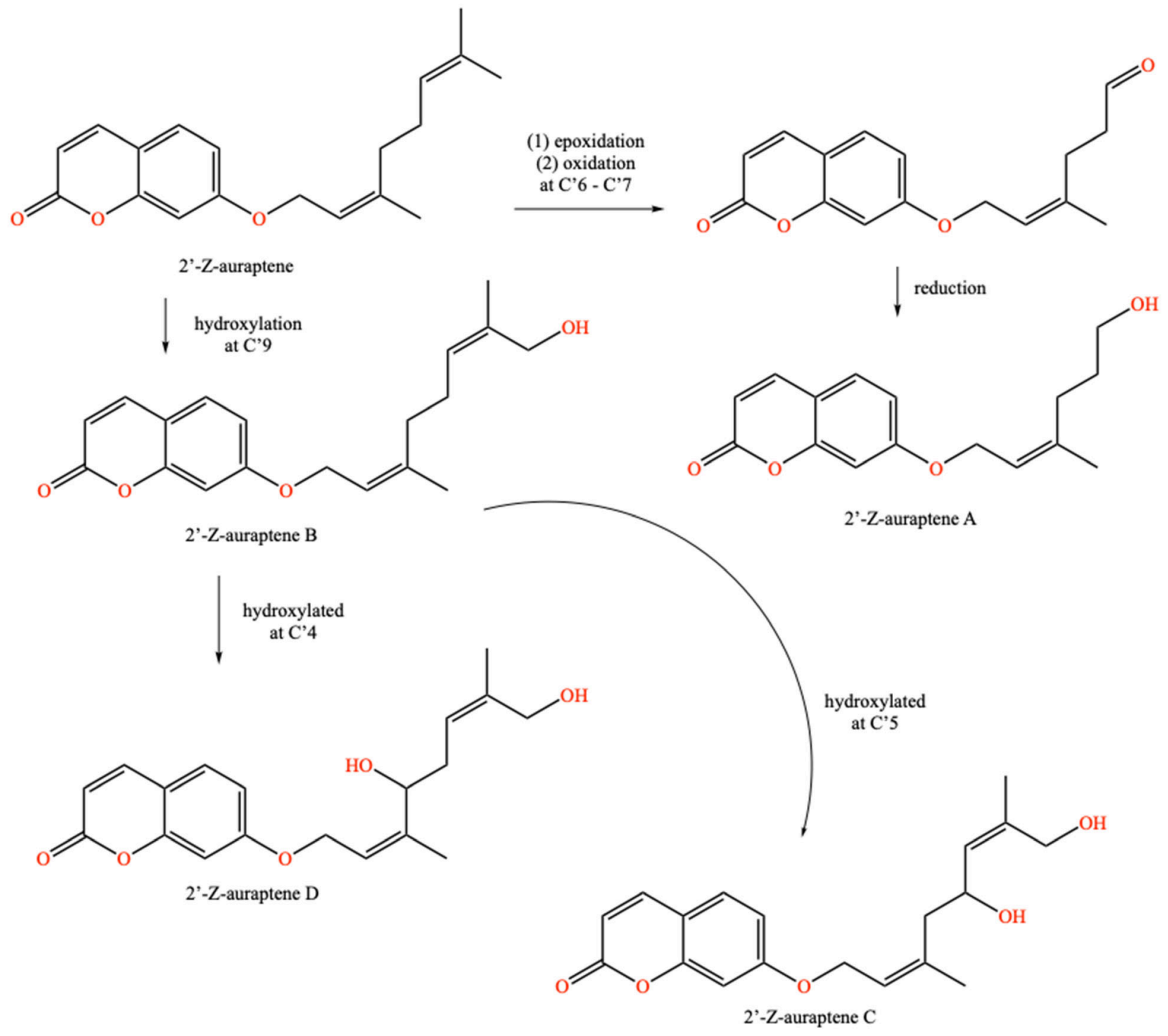
Biotransformation of terpene coumarin by *Mucor polymorphosporus*.

Subsequently, the microbial transformation of the terpene coumarin 2′-Z auraptene was conducted employing the fungus *Mucor polymorphosporus*, producing four novel derivatives. These four terpene coumarins were named as 2′-Z auraptene A, 2′-Z auraptene B, 2′-Z auraptene C, and 2′-Z auraptene D, each of them possesses unique structural modifications, and the chemical names are as follows: (Z)-7-((3′,7′-dimethylocta-2′,6′-dien-1′-yl)oxy)-2H-chromen-2-one; 7-(((2′Z,6′Z)-9′-hydroxy-3′,7′-dimethylocta-2′,6′-dien-1′-yl) oxy)-2H-chromen-2-one; 7-(((2′Z,6′Z)-4′,9′-hydroxy-3′,7′-dimethylocta-2′,6′-dien-1′-yl)oxy)-2H-chromen-2-one; and 7-((2′Z,6′Z)-5′,9′-hydroxy-3′7′-dimethylocta-2′,6′-dien-1′-yl)-2H-chromen-2-one, respectively. The conversion of parent compound, cis-auraptene, into these four derivatives by *Mucor polymorphosporus* is a complex mechanism involving oxidation, hydroxylation, and reduction reactions. The mechanism is initiated by an epoxidation reaction occurring at the C-6′ and C-7′ olefinic bonds of the terpene side chain. Following this epoxidation reaction, a C=C bond cleavage process takes place, resulting in the formation of a carbonyl group at the C-6′ position. Moreover, the formation of the carbonyl group was followed by a reduction process to yield 2′-Z auraptene A. Subsequently, 2′-Z auraptene B is formed via a hydroxylation specifically at the C-9′ position. This C-9′ hydroxylated intermediate then acts as a precursor for the subsequent derivatives, undergoing further hydroxylation at the C-4′ or C-5′ positions to yield 2′-Z auraptene C and 2′-Z auraptene D, respectively. The schematic reaction is shown in Figure 11. The selective biotransformation capability of this fungus strains gives an essential advantage of microbial biocatalysis. This fungus can selectively introduce a hydroxyl group at the C-9′ position of the side chain, which provides a new hydroxylating route for cis-auraptene, which is challenging to achieve using conventional chemical synthetic reactions. Microbial transformation systems offer precise levels of regioselectivity and stereoselectivity under mild conditions, serving as crucial tools for accessing difficult synthesize reaction of natural product analogues, such as the bioactive compound 2′-Z auraptene derivatives [46].

### 2.3. Methylation and Demethylation of Coumarins: Modulation of Polarity, Stability, and Biological Activity

The microbial transformation of coumarins relies on the enzymatic modification pathway, which particularly changes the polarity and lipophilicity of the derivative compounds. The changing of the polarity is often achieved by the addition of a hydroxyl (-OH) group, which increases the polarity of the compounds. However, the methylation of the hydroxyl group led to the methoxylated coumarins, such as scopoletin and xanthotoxin, which decreases the polarity but enhances lipophilicity. The increase of lipophilicity enhances membrane permeability, cellular uptake, and bioactivity. It also improves chemical stability by shielding the hydroxyl group from chemical and enzymatic degradation [47]. On the other hand, this process can undergo oxidative O-demethylation, a reaction that replaces the methoxy group (-OCH_3_) in the coumarin molecule with a hydroxyl group, releasing formaldehyde in the process. By shifting the hydroxyl group, O-demethylation significantly enhances the polarity of the coumarin derivatives, thereby increasing the biological properties [48]. Furthermore, methylation and demethylation are principal phase I and phase II metabolic reactions to create the biological activity of coumarin derivatives. The dealkylation step is particularly an oxidative phase I reaction that removes an alkyl group from an oxygen atom, converting an ether into a more polar phenolic hydroxyl group (e.g., deethylation of 7-ethoxy coumarin into 7-hydroxycoumarin). On the other hand, methylation is a phase II conjugative reaction that adds a methyl group to a hydroxyl group, altering a phenol back into a less polar methoxy ether [49]. In bacteria, demethylation of coumarins is facilitated by CYP450 monooxygenases. These enzymes work by introducing an oxygen atom into the methyl group, then it attaches to the coumarin ring, forming the hydroxymethyl intermediate. Then, the oxidation process leads to the release of formaldehyde and the formation of a hydroxyl group at the position where the methyl group was located. This demethylation process, followed by the oxidative reaction, increases the hydrophilicity of the coumarin derivative molecules, especially the bioavailability and the interaction with the biological targets [50]. Similarly, fungi also have an important role in the demethylation process of coumarins. Various types of fungi, including *Streptomyces avertimilis*, *Streptomyces griseus*, and *Candida tropicalis,* consist of enzymatic systems that facilitate O-demethylation [48].

**Figure 12:**
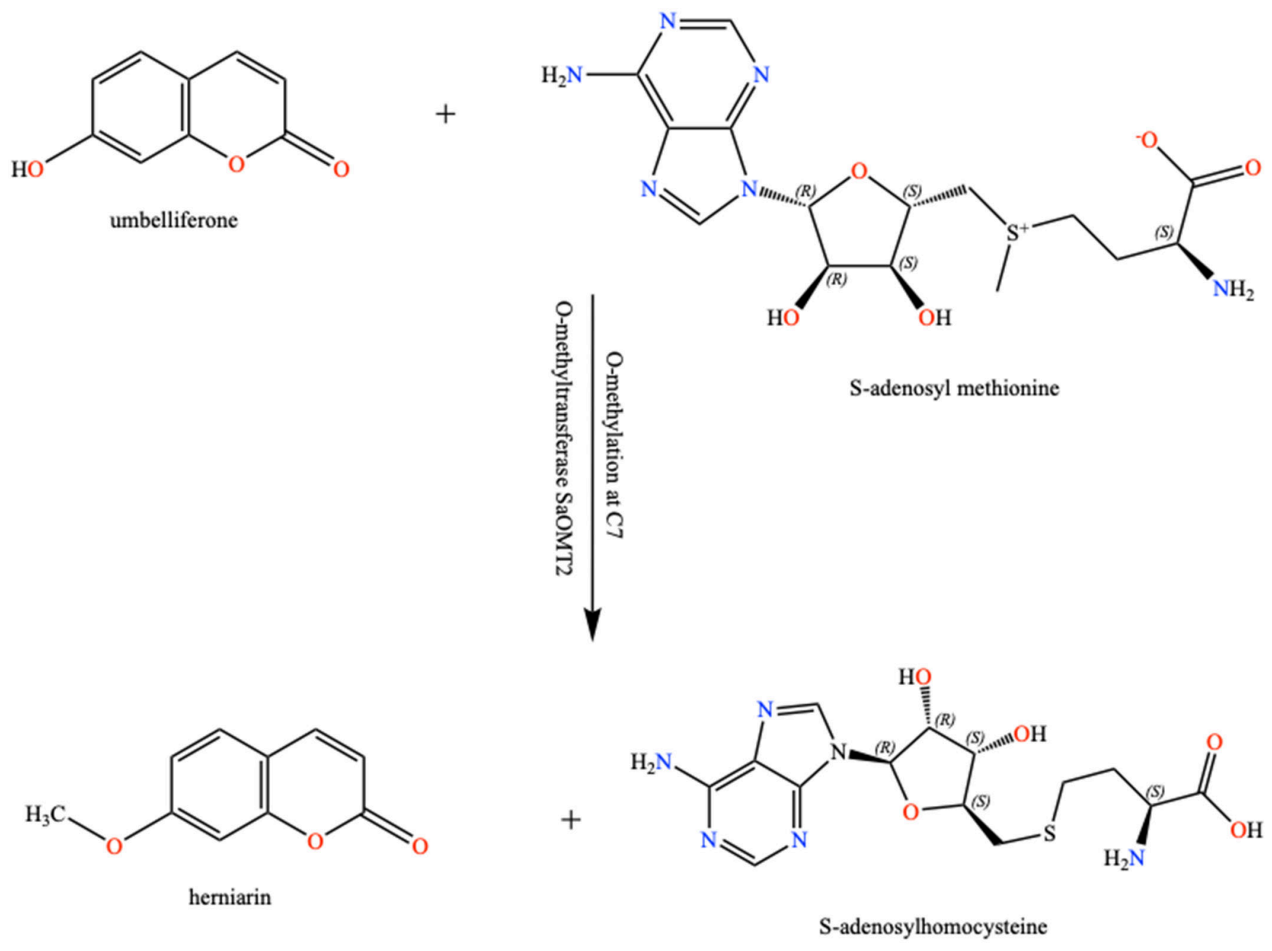
SaOMT2-catalyzed regiospecific O-methylation of umbelliferone using SAM produces the coumarin derivative, herniarin.

As previously mentioned, the microbial transformation of specific coumarin derivatives, including esculetin, skimming, and herniarin, is achieved via both enzymatic catalysis and whole-cell microbial transformation. The essential steps in this pathway are O-hydroxylation and lactonization. Specifically, the biosynthesis of herniarin involves a specific methylation reaction. The biotransformation of this compound focuses on the methylation of hydroxylated coumarin, umbelliferone. Herniarin production is catalyzed by the O-methyltransferase SaOMT_2_ from *Streptomyces avertimilis*, which facilitates the O-methylation of umbelliferone, SaOMT_2_ belongs to the S-adenonysl methionine-dependent (SAM-dependent) methyltransferase superfamily, an enzyme class responsible for adding methyl groups to oxygen atoms. This modification often increases the stability and lipophilicity of the derivative compounds. This reaction requires two molecules: SAM, which donates the activated methyl group, and umbelliferone, which accepts the methyl group at the C-7 hydroxyl position. This mechanism begins when SAM and umbelliferone bind to the enzyme’s catalytic site and then precisely attack the activated methyl group on SAM at the C-7 position. This mechanism results in the formation of an ether bond, producing the final product, herniarin, followed by the release of S-adenosyl-L-homocysteine (SAH). The methylation catalyzed by SaOMT_2_ is a regiospecific reaction, which means the enzyme specifically targets and methylates only the hydroxyl group at the C-7 position of the coumarin ring, as shown in Figure 12. This characteristic of the enzymatic reactions is essential because the pattern of methylation will result in distinct biological properties [51].

**Figure 13:**
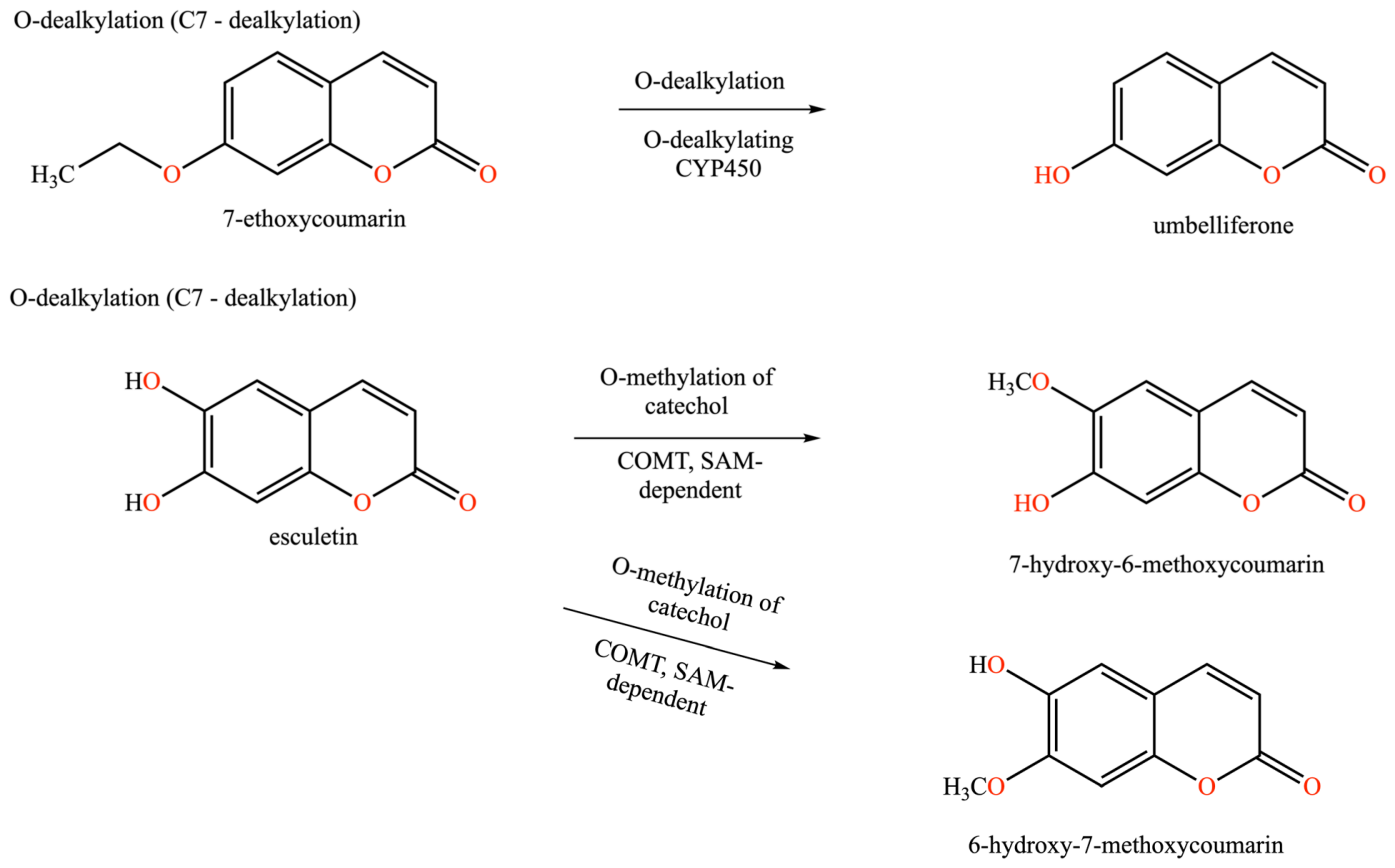
The O-dealkylation and O-methylation reactions in the biotransformation of 7-ethoxycoumarin by *Streptomyces griseus*.

In the biotransformation of 7-ethoxycoumarin by *Streptomyces griseus,* there are two different alkylation processes involved: the dealkylation reaction, particularly de-ethylation, and the methylation reaction. The first reaction is the O-deethylation of 7-ethoxycoumarin, which is carried out by removing the ethyl group attached to the oxygen atom at the C-7 position and converting an ether into a phenolic hydroxyl group. This step produces the intermediate compound, 7-hydroxycoumarin, and acetaldehyde as the side product, since the cleaved ethyl group is commonly oxidized and released as an aldehyde. This O-deethylation reaction is particularly catalyzed by CYP450 monooxygenases, which require O_2_ molecule and an electron donor such as NADPH to perform the reaction [52,53]. After the O-deethylation process in the microbial transformation of 7-ethoxycoumarin, the aromatic hydroxylation process results in the dehydroxylated intermediate compound, 6,7-dihydroxycoumarin, which was already mentioned in Section 2.2. Further, the last stage in this biotransformation process by *Streptomyces griseus* is the O-methylation of the 6,7-dihydroxycoumarin to produce the monoethoxy derivative, 7-hydroxy-6-methoxycoumarin, as depicted in Figure 13. This reaction occurs by the addition of a methyl group to an oxygen atom, which is catalyzed by catechol-O-methyltransferase (COMT), a type of class I SAM-dependent O-methyltransferase enzyme. SAM also acts as the methyl donor for the reaction, resulting in the conversion of SAM into SAH. COMT is involved in transferring a methyl group from SAM to one of the hydroxyl groups in the 6,7-dihydroxycoumarin. This enzyme works by binding to 6,7-dihydroxycoumarin and SAM, and the mechanism is a typical S_N_2 substitution reaction that occurs when the nucleophilic attack has occurred, where the oxygen atom of the hydroxyl group attacks the electrophilic methyl group of SAM, yielding the release of SAH and the methylated product [52,54].

**Figure 14:**
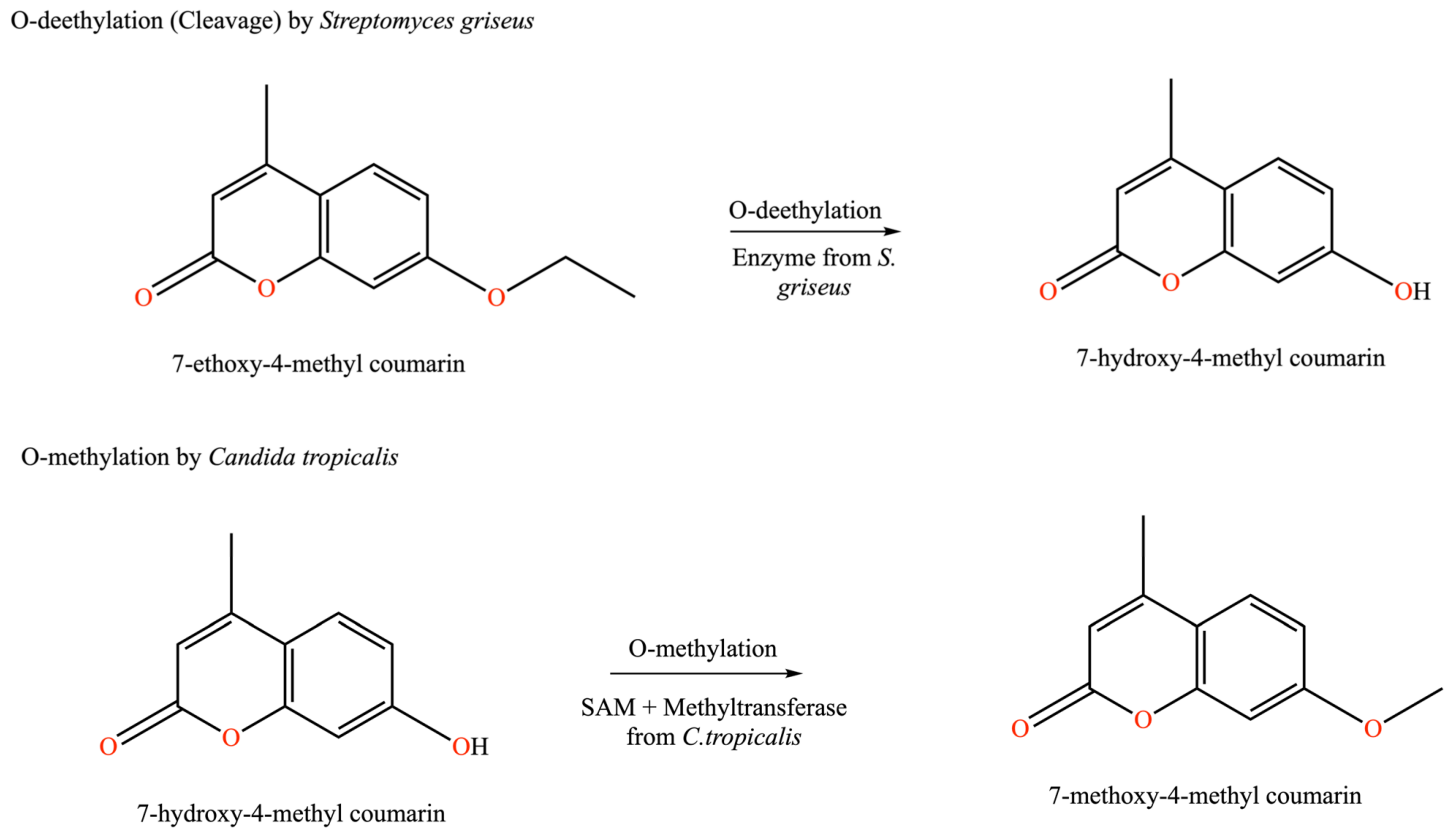
Sequential biotransformation of coumarin derivative involves O-deethylation by *Streptomyces griseus* followed by the O-methylation of the resulting precursor by *Candida tropicalis*.

The biotransformation performed by *Streptomyces griseus* and *Candida tropicalis* is a sequential stage in the microbial synthesis pathway for the transformation of coumarin derivatives to produce different bioactive coumarin derivatives. The relationship between the two reactions is that the product of the O-dealkylation, which is catalyzed by *Streptomyces griseus,* serves as a substrate for the biotransformation step carried out by *Candida tropicalis*. The initial step involves *Streptomyces griseus* on 7-ethoxy-4-methylcoumarin. This microorganism conducts O-deethylation, which involves the removal of the ethyl group from the ether linkage at the C-7 position and replaces it with a hydrogen to produce 7-hydroxy-4-methylcoumarin. Thus, this product, 7-hydroxy-4-methylcoumarin from previous steps, constitutes the precursor for O-methylation for *Candida tropicalis*. This yeast strain performs the biotransformation via a reaction. In this reaction, the OH group at the C-7 position of 7-hydroxy-4-methylcoumarin is methylated, resulting the corresponding methyl ether derivative, 7-methoxy-4-methylcoumarin as shown in Figure 14 [47].

In addition, the sequential process of microbial transformation on 7-(3-(cyclopropylamino)-2-hydropropoxy)-4-methyl-2H-chromen-2-one was employed by using free cells of *Candida albicans* to produce 7-(3-(cyclopropylamino)-2-hydroxypropoxy)-4-methoxymethyl-chromen-2-one or 7-(3-cyclopropylamino-2-methoxy-propoxy)-4-hydroxymethyl-chromen-2-one. This microbial transformation is driven by oxidation followed by a methylation process, facilitated by enzymes produced during the metabolic activities of *C. albicans* cells [55,56].

Beyond the single-strain reactions described above, synthetic biology efforts have shifted to coupling biocatalyst systems to engineered strains or heterologous hosts rather than depending on conventional fermentations. Division of co-culture systems, where the complementary catalytic strains are combined, has been successfully applied to optimize metabolic pathways, enhance substrate conversion efficiently, and cultivate bioproduction capabilities compared to monocultures. These mechanisms have already been engineered for phenypropanoid substrates related to the coumarin pathway through synthetic co-cultivation by genetically engineered *Bacillus substilis* to secrete specific enzymes to release hydroxycinnamic acids. Then, these hydroxycinnamic acids were converted into phenylpropanoids through biotransformation using engineered *Escherichia coli* [57]. Similarly, engineered *E. coli* pathways combining TAL/4CL/HCT/CCoAOMT/C3H-type enzymes to provide de novo biosynthesis of scopoletin and umbelliferone. Basically, the generation of umbelliferone is also achieved by using a synthetic biology system based on microbial cells that produces coumarins from tyrosine, utilizing *Rhodoturula glutinis* to secrete tyrosine ammonia lyase to cut the mechanism of cinnamic acid 4-hydroxylase, reflecting that heterologous mechanisms with engineered hosts can replace multi-organism series fermentations for O-methylated/O-demethylated coumarin precursors [58].

Moreover, protein engineering is starting to manage the regioselectivity limitations of methyltransferases and CYP450 demethylases discussed above. For instance, non-natural and site-directed mutagenesis methods used to structurally modify P450 monooxygenases have shown that active-site mutations can result in large shifts in regioselectivity. Furthermore, semi-mutagenesis of the biosynthesis of P450 enzymes has been utilized to modify the reactivity and selectivity of the biocatalysts to alter analog reactions of coumarin hydroxylation and demethylation. Thus, modifying the active site of CYP450 demethylase and SAM-dependent O-methyltransferases resulted in the capabilities of these enzymes for coumarin transformation. Moreover, these approaches can be used with either a series or a group of different microbes, or a single engineered microbe can be applied for moving the methylation or demethylation microbial transformation from laboratory-scale fermentation toward an industrially scalable platform [59].

### 2.4. Glycosylation and Other Tailoring Processes: Improving Solubility and Modulating Bioactivity

Glycosylation in microbial transformation of coumarins is an essential mechanism that serves to convert the physicochemical and biological characteristics of secondary metabolites of coumarins. This process is particularly mediated by plant enzymes called UDP-glycosyltransferases (UGTs), which belong to the glycosyltransferase family 1 (GT1). This family of enzymes utilizes an activated sugar donor, commonly UDP-glucose. The UGT enzymes carried out a regioselective transfer of the sugar molecule to the coumarin skeleton to form an O-glycoside via linkage to a hydroxyl group. In a few pathways, C-glycosyltransferase (CGTs) form more stable C-glycosides through a direct carbon-carbon bond, usually at C-6 and C-8. This modification is essential to increase the solubility of the hydrophobic coumarins, which is important for overall bioavailability, leading to an enhancement of antioxidant or anti-inflammatory properties [56,60,61].

The transformation of umbelliferone to skimmin is an example of highly selective O-glycosylation catalyzed by uridine diphosphate glycosyltransferase YjiC from non-pathogenic *Bacillus licheniformis*. This biotransformation involves the glycosylation of umbelliferone at the C-7 position to produce skimmin [51]. Moreover, YjiC from non-pathogenic *B. licheniformis* DSM 13 strain is a versatile glycosyltransferase with the ability to catalyze nucleophilic glycosylation not only at hydroxyl groups (O-glycosylation), but also at amino groups (N-glycosylation) and thiol groups (S-glycosylation). YjiC enzyme shows important flexibility toward its glycosyl donors, by efficiently transferring sugar molecules from a wide range of nucleotide carriers. This donor promiscuity is highlighted by its ability to utilize UDP-α-D-glucose, UDP-N-acetylglucosamine, UDP-N-acetyl-galactosamine, and UDP-α-D-glucuronic acid. On the acceptor side, YjiC has the capability to glycosylate compounds containing amino (-NH_2_) and thiol (-SH) groups, such as 3,4-dichlorobenzenethiol and 3-aminocoumarin [62].

**Figure 15:**
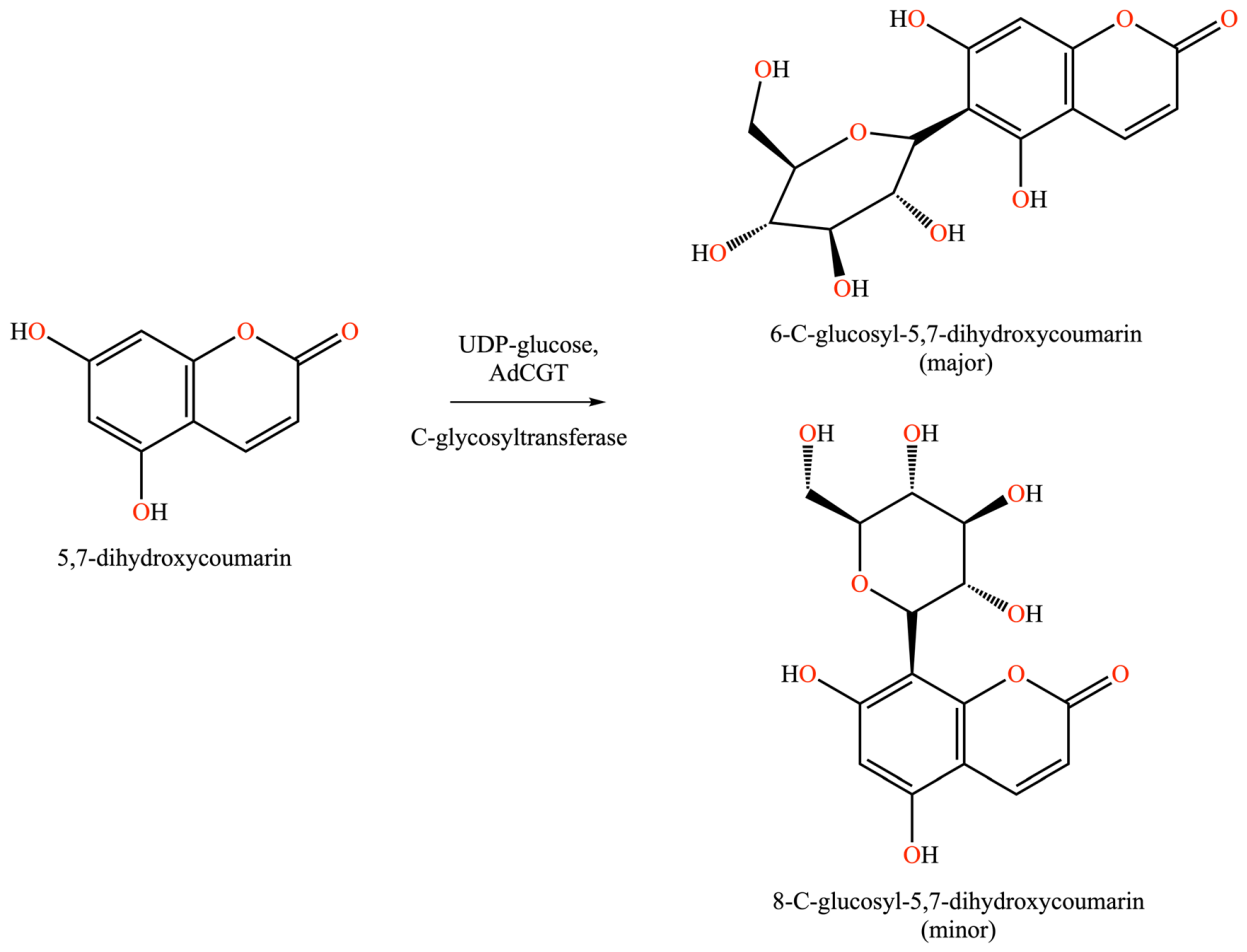
C-glycosylation of 5,7-dihydroxycoumarin by the glycosyltransferase AdCGT using UDP glucose, producing dauroside D(6-C-glycosyl-5,7-dihydroxycoumarin) as a major product and a minor C-8 isomer.

The gene glycosyl transferase, isolated from *Angelica decursiva,* was cloned and constructed in an *E. coli* heterologous expression system and showed C-glycosyl transferase activity by in vitro activity assay using 5,7-dihydroxycoumarin as substrate. As a result, a C-glycosylated product at position C-8 along with the main product at the C-6 position was obtained typically in a defined ratio depending on substrate orientation within the enzyme’s active site. Unlike the more common O-glycosylation observed with many phenolic compounds, the enzyme transfers a glucose moiety from UDP-glucose directly onto the aromatic ring of the coumarin scaffold, forming a stable C–C bond (Figure 15) [56].

**Figure 16:**
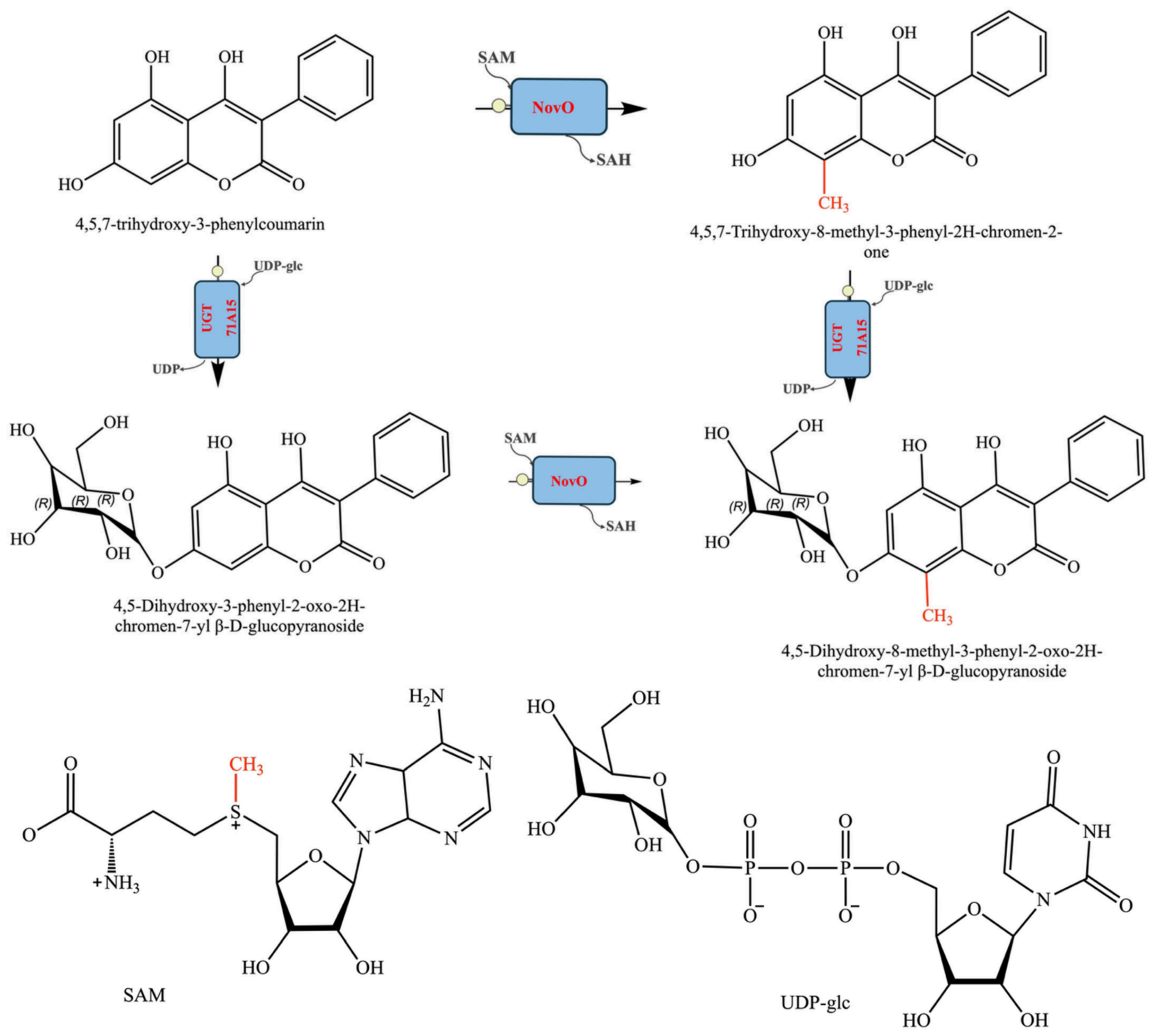
Possible pathways of enzymatic methylation and glycosylation.

In other studies, a polyhydroxylated coumarin was used as a model substrate for biotransformation with two combined enzymes: a SAM-dependent C-methyltransferase (NovO, from *Streptomyces*) and a UDP-glucose-dependent glycosyltransferase (UGT71A15, from apple) (Figure 16). The glycosylation step was highly regioselective, producing exclusively the 7-O-β-D-glucoside, despite the presence of multiple hydroxyl groups, demonstrating excellent control over site selectivity [63].

**Figure 17:**
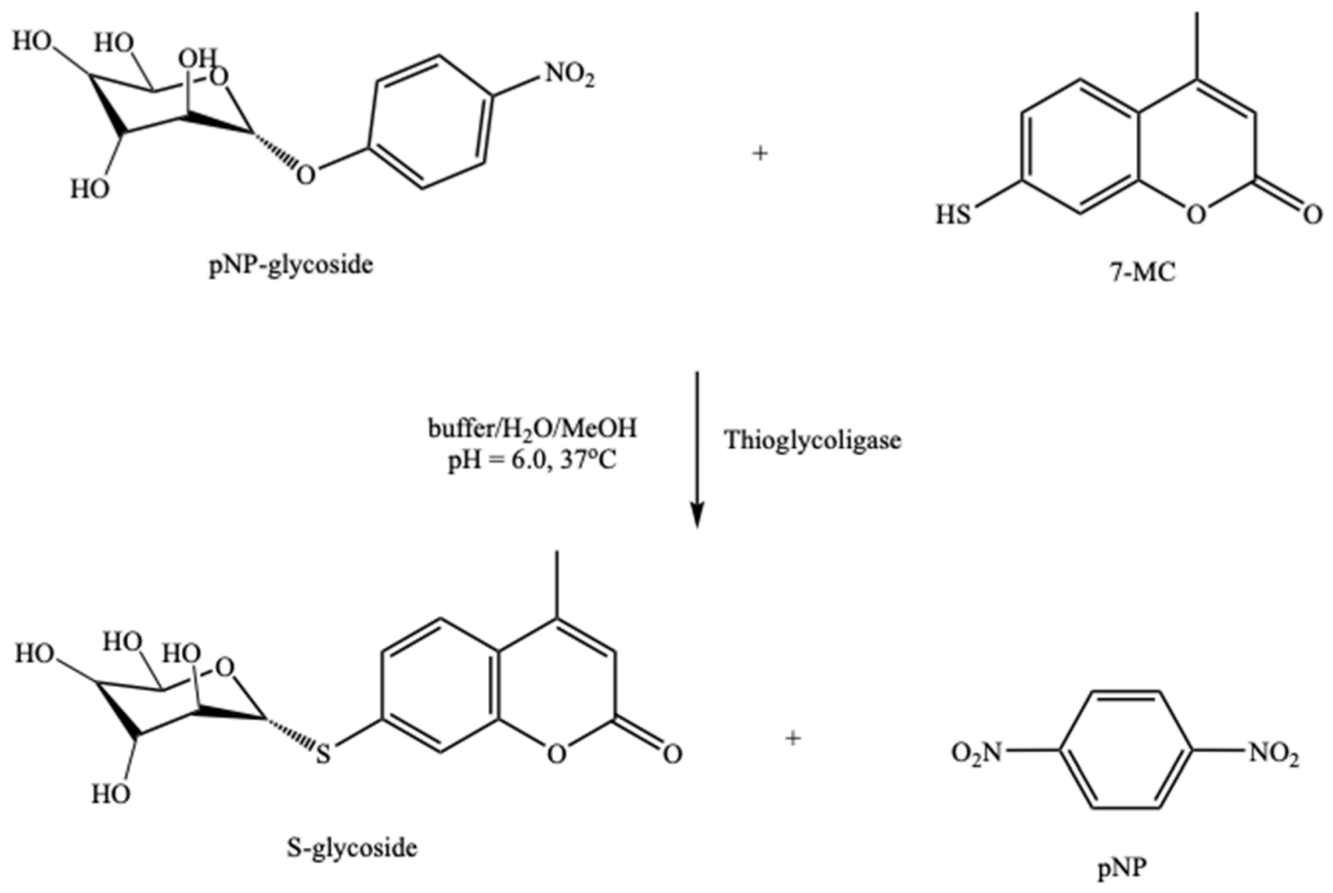
Possible scheme of thioglycosylation.

Another novel enzymatic strategy was applied to obtain S-glycosylated coumarins using engineered glycosidases (thioglycoligases). A sulfur-functionalized analogue of umbelliferone (7-mercapto-4-methylcoumarin) was used as the glycosyl acceptor, enabling glycosylation through a thiol group at position 7. Unlike classical O-glycosylation of hydroxycoumarins, S-glycosylation (formation of C–S–glycosidic bonds) was achieved by coupling activated sugar donors (p-nitrophenyl glycosides) with the thiol-containing coumarin in a one-pot enzymatic reaction. The resulting S-glycosylated coumarins showed enhanced stability compared to O-glycosides due to the resistance of C–S bonds to hydrolysis and exhibited improved fluorescence properties, particularly in aqueous media, making them suitable as probes. These fluorescence properties were experimentally verified rather than inferred; the resulting S-glycosylated coumarins were determined by UV-Vis and fluorescence spectroscopy both in organic solvent (N,N-dimethylformamide-DMF) and under aqueous physiological conditions (PBS:DMF, 99:1), which allowed comparison of their optical characteristics in biological systems. This approach showed a difference between the polarity of the ground and excited states of the S-glycosides. Importantly, the improved fluorescence retention in aqueous systems and the panel of compounds were examined to be non-cytotoxic across human cell lines, distinguishing them from many native coumarins and supporting their use in biomedical imaging and sensing applications (Figure 17) [62].

Coumarin and 8-hydroxycoumarin were reduced by xenobiotic reductase XenA. XenA catalyzes the NADH/NADPH-dependent reduction of 8-hydroxycoumarin, a known intermediate in the degradation of quinoline by *Pseudomonas putida* 86. This XenA is classified as a flavin-containing enzyme and belongs to the family of Old Yellow Enzyme (OYE). This family of enzymes is known as flavoprotein enzymes, which catalyze the stereoselective reduction of α,β-unsaturated carbonyl compounds. XenA plays an important role in the catabolism process of coumarin and its derivatives, such as 8-hydroxycoumarin. This enzyme catalyzes the reduction process of the C3-C4 α,β-unsaturated double bond in the lactone ring. The conversion of 8-hydroxycoumarin to yield 8-hydroxy-3,4-dihydrocoumarin commonly starts with the microbial degradation reaction of coumarins, resulting in the hydrolysis reaction of the lactone ring to form the phenolic propionic acid derivative, such as melilotic acid. Particularly, XenA uses NADPH as a reducing cofactor, but sometimes NADH can be utilized too. The initial reaction starts when the oxidized enzyme binds the reduced structure of the NADPH cofactor; then the hydride ion is transferred from the C-4 position of NADPH to the N-5 position of the flavin mononucleotide (FMN). This pathway reduces the cofactor to FMNH_2_ and releases the oxidized NAD(P)^+^ [64].

Coumarin carboxamide is one of the essential coumarin derivatives because of its biopharmaceutical properties; for instance, it inhibits plasmatic-activated factor VII and is used as an anticoagulant drug. Chidamide and entinostat are histone deacetylase inhibitors that have been approved by the FDA to treat lymphoma or myeloma. The synthesis of coumarin carboxamide via conventional reaction has several problems, including a multistep process and the use of harsh chemical catalysts [65,66,67]. Therefore, biocatalysis offers a green reaction by using enzymes like lipase PPL and the coumarin C-glucosyltransferase MaCGT. In addition, the enzymatic synthesis of coumarin carboxamide compounds is a two-step mechanism. The first stage is the intermediate formation; the salicylaldehyde derivatives and dimethyl malonate are reacted to produce the intermediate compounds, coumarin carboxylate methyl derivatives. This reaction is co-catalyzed by the enzyme and potassium carbonate. The second stage is the reaction between coumarin carboxylate methyl and several amines such as benzylamine, isobutylamine, and 4-methoxybenzylamine, which is catalyzed by lipozyme TL IM from *Thermomyces lanuginosus* through continuous-flow microreactors. This enzymatic reaction involves a transesterification and/or amidation of the C3 methyl ester with the amine, resulting in coumarin carboxamide derivatives [68].

## 3. Pharmaceutical Potential of Coumarins

### 3.1. Pharmaceutical Properties of Coumarins

**Figure 18:**
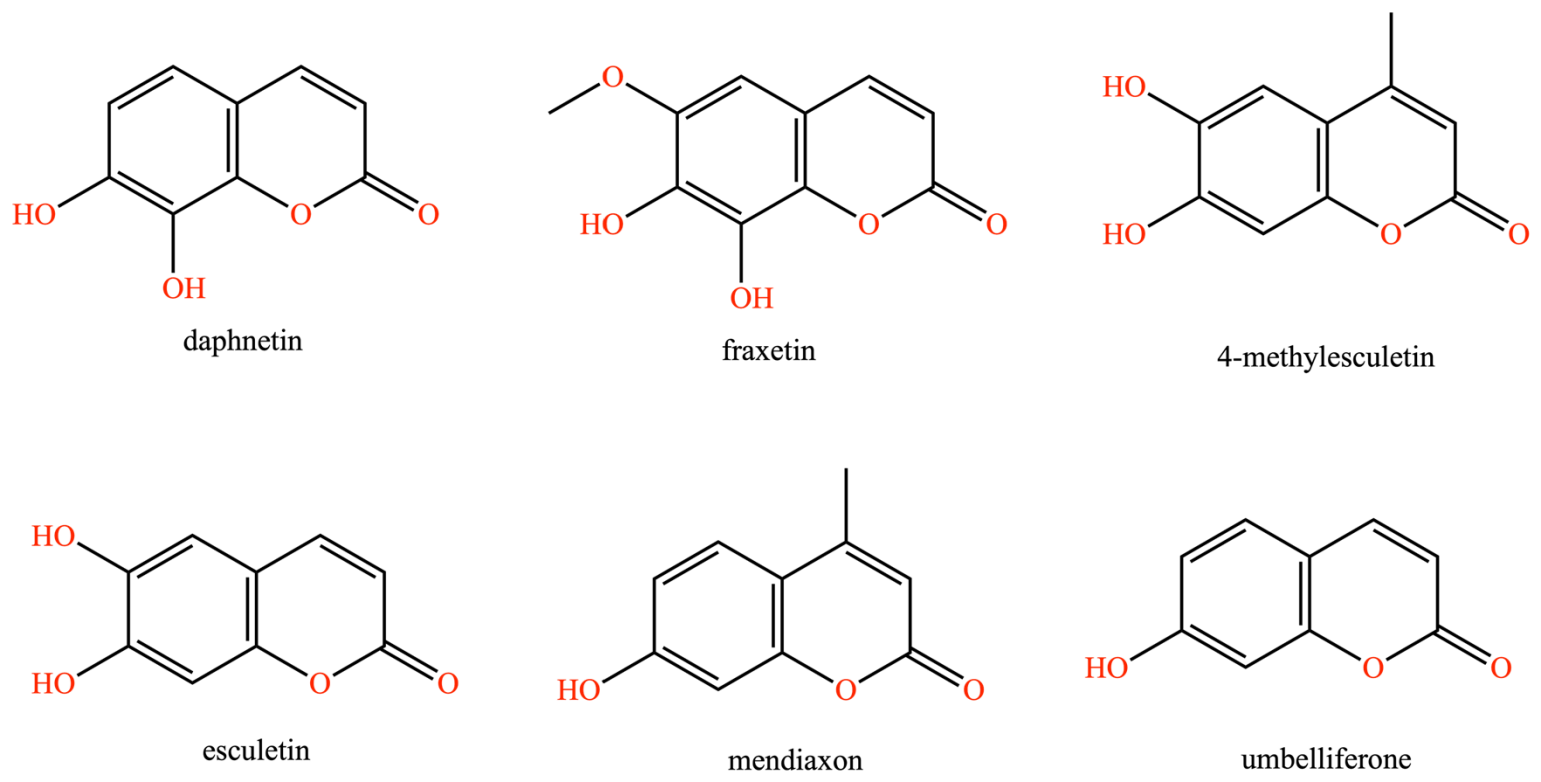
Chemical structures of simple coumarins.

The pharmacological properties, coumarins remain an important point of research due to the diverse substitution patterns possible on the benzopyrone core, with the basic structural motifs of simple coumarins displayed in Figure 18. This chemical flexibility facilitates the transition from natural leads to high-potency analogues [69,70,71,72]. Simple hydroxy- and methoxy-substituted coumarins often modulate redox and inflammatory pathways, whereas more complex scaffolds engage discrete enzymatic targets, including viral proteases/replicases and mammalian enzymes such as steroid 5α-reductase; in parallel, the vitamin K antagonist (VKA) subgroup exemplifies how coumarin cores can be clinically leveraged through precise interference with hepatic γ-carboxylation of coagulation factors [69,70,71,73,74,75,76]. Accordingly, this overview synthesizes therapeutic themes, representative molecules, and safety considerations, while situating preclinical signals within translational contexts.

Early surveys of simple coumarins as antioxidants and radical scavengers show molecules such as esculetin and umbelliferone attenuate reactive oxygen species and remodel redox-sensitive transcriptional programs, thereby providing a biochemical rationale for their anti-inflammatory effects observed with daphnetin, fraxetin, and 4-methylesculetin in cellular and animal models [69,70,71]. Moreover, several coumarin scaffolds have demonstrated in vitro activity against hepatitis C virus via interference with viral protease or replicase machinery, although rigorous translational validation is still required before clinical extrapolation can be justified [70,71,72]. In addition, selected derivatives inhibit steroid 5α-reductase in biochemical and cellular systems, suggesting potential applications in androgen-driven conditions that merit targeted medicinal chemistry and pharmacodynamic confirmation [70,71].

**Figure 19:**
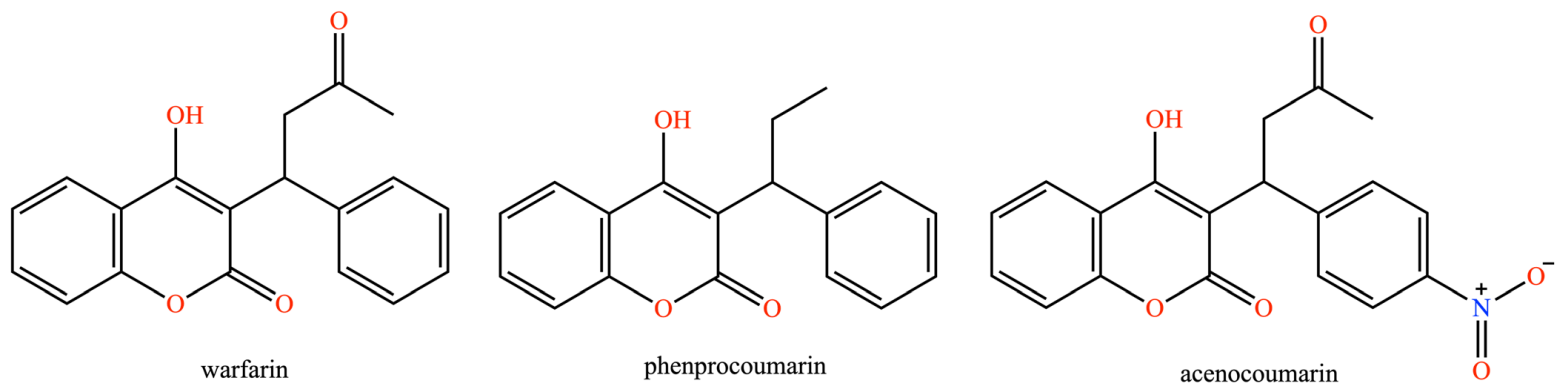
Chemical structures of anticoagulant coumarins.

Anticoagulant coumarins (vitamin K antagonists), including warfarin, phenprocoumon, and acenocoumarol, whose structures are depicted in Figure 19, as delineated in clinical pharmacology and guideline literature, act through inhibition of vitamin K epoxide reductase complex subunit 1 (VKORC1), thereby impairing vitamin K–dependent γ-carboxylation of factors II, VII, IX, and X as well as proteins C and S. The resulting undercarboxylated proteins are functionally inactive, which suppresses thrombin generation and the broader coagulation cascade [24,25,26,27,28,29,30].

Given their narrow therapeutic indices and substantial interindividual variability, dosing is individualized using the international normalized ratio, with established public health relevance underscored by warfarin’s listing on the World Health Organization (WHO) model list of essential medicines [75,77,78]. Notably, warfarin is administered as a racemate wherein the S-enantiomer is approximately 2–5 times more potent than the R-enantiomer and is primarily cleared by CYP2C9, whereas the R-enantiomer is metabolized mainly by CYP1A2 and CYP3A4. Similarly, phenprocoumon is also administered as a racemate and exhibits pharmacokinetics influenced by CYP3A4 with contribution from CYP2C9. In contrast, acenocoumarol is shorter-acting than warfarin and phenprocoumon, a characteristic that facilitates perioperative management but often necessitates more frequent dose adjustments, with CYP2C9 playing a key role in hepatic clearance [73,75,76,79,80,81]. In this context, half-life differences are clinically significant, with approximate averages near 40 h for warfarin and around 150 h for phenprocoumon, while acenocoumarol displays the shortest effective half-life among the three, acknowledging wide interindividual ranges [75,80].

**Figure 20:**
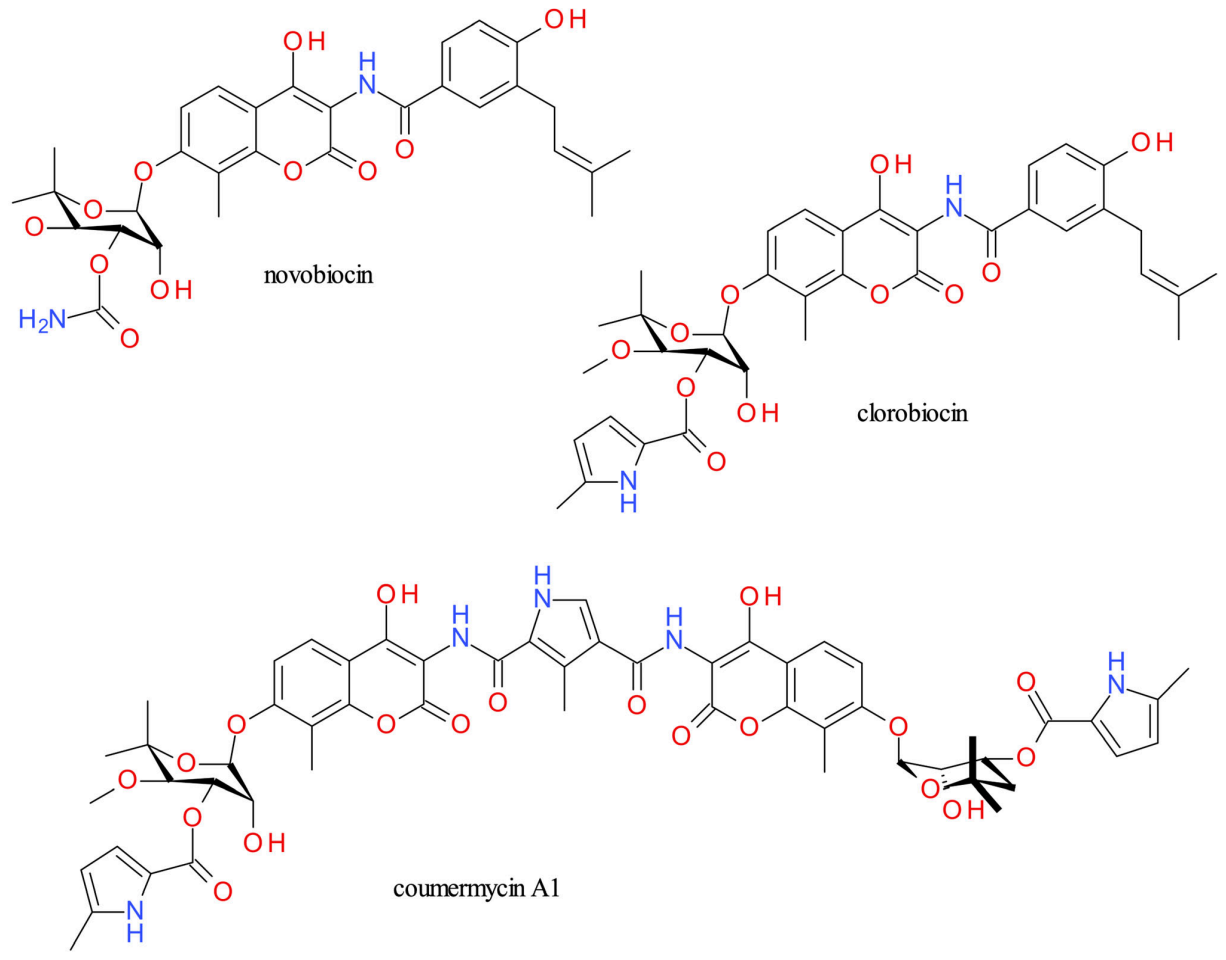
Chemical structures of antimicrobial aminocoumarins.

Furthermore, clinically important drug–drug and drug–food interactions exemplified by azole antifungals, macrolides, fluoroquinolones, amiodarone, non-steroidal anti-inflammatory drugs (NSAIDs), variable dietary vitamin K, and alcohol necessitate vigilant monitoring because of bleeding risks, particularly when NSAIDs or antiplatelets are co-administered [77,79]. Converging evidence from the Clinical Pharmacogenetics Implementation Consortium (CPIC) and large cohort analyses indicates that CYP2C9 and VKORC1 genotypes influence warfarin and acenocoumarol dose requirements and bleeding risk and that genotype-guided dosing can improve the time in therapeutic range, thereby enhancing safety and effectiveness [73,76,81]. In contrast to antagonists of vitamin K (VKAs), aminocoumarin antibiotics shown in Figure 20, such as novobiocin, clorobiocin, and coumermycin A1, act predominantly by binding the ATPase site of the DNA gyrase subunit B (gyrB) subunit of bacteria, with potential effects on topoisomerase IV, leading to inhibition of DNA replication and transcription [82,83,84,85].

While these agents display potent in vitro activity against many gram-positive organisms, clinical deployment has been curtailed by toxicity, limited efficacy relative to contemporary antibiotics, and resistance; consequently, they now serve primarily as biochemical tools and probes to study type II topoisomerases and ATP-binding enzyme mechanisms [82,86]. Originally, novobiocin was isolated from *Streptomyces niveus* and was explored for staphylococcal infections, whereas clorobiocin and the dimeric coumermycin A1 have contributed to mechanistic and structural biology insights despite constraints such as hepatotoxicity and poor bioavailability [82,87,88,89].

**Figure 21:**
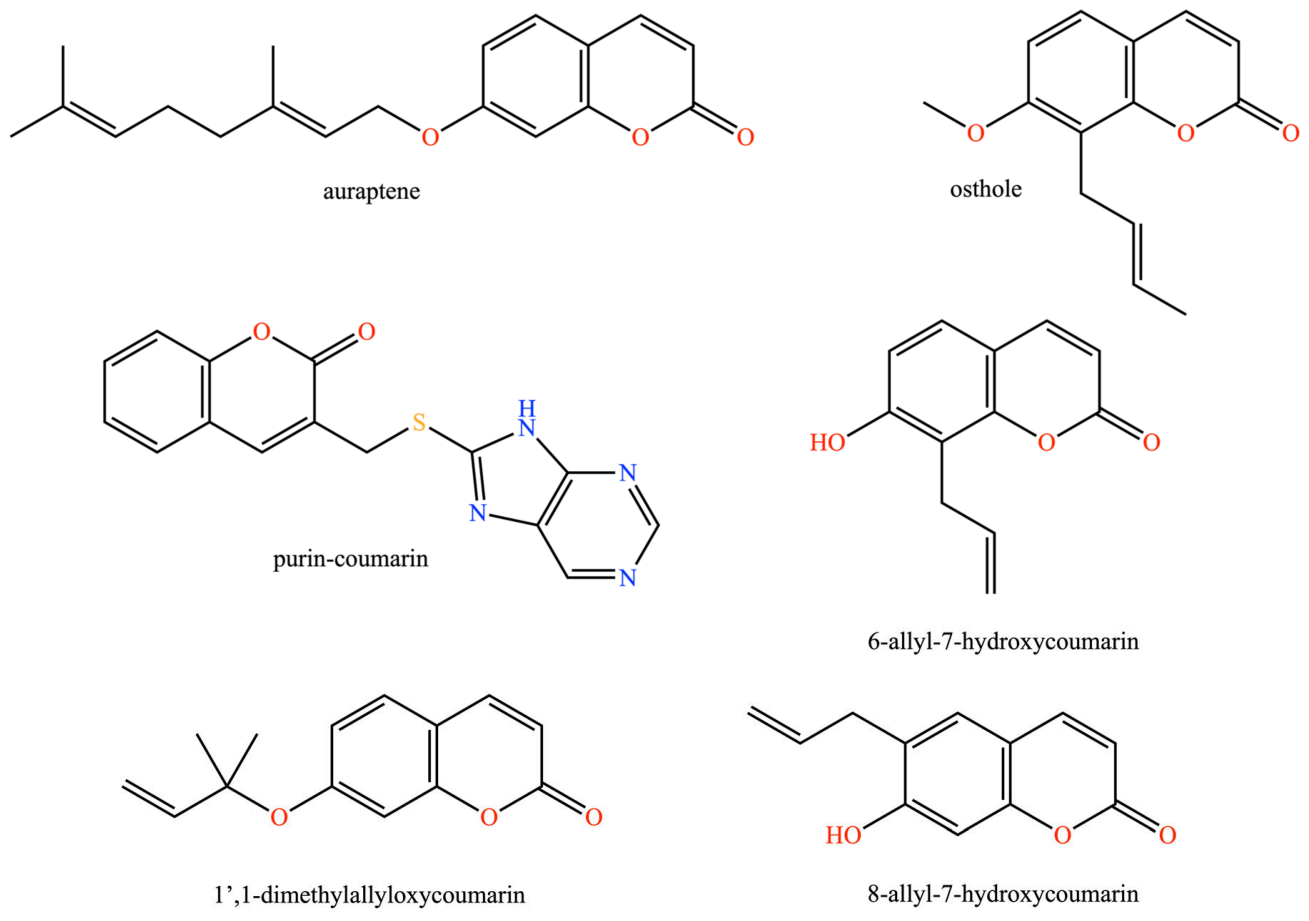
Chemical structures of pharmacologically significant natural coumarins.

In antibacterial applications, natural coumarins have potential for their biological activities, particularly in oncology. Building on substantial preclinical research, osthol (osthole), a prenylated simple coumarin found in *Cnidium monnieri* and *Angelica* species, exhibits anti-inflammatory, antimicrobial, vasodilatory, neuroprotective, and anticancer effects mediated through induction of apoptosis, cell-cycle arrest, and modulation of oncogenic signaling pathways. The chemical structures of several pharmacologically significant coumarins are illustrated in Figure 21; however, these compounds remain under investigation and are not yet approved for clinical use [70,71,90].

In parallel, auraptene, aprenyloxycoumarin abundant in citrus fruits and conveniently accessible from umbelliferone and geranyl bromide using efficient, green, chromatograph-free synthesis, shows robust anti-inflammatory and antioxidant properties in preclinical systems and has garnered interest for anticancer potential [69,70,71]. Significantly, in colorectal cancer models, auraptene reduces cancer stem cell markers such as CD44 and CD166 in drug-resistant cells and impairs spheroid and clonogenic growth; mechanistic readouts are consistent with preventing the generation of cancer stem cells rather than exerting direct cytotoxicity against established cancer stem cell populations, a feature that could mitigate chemoresistance and relapse pending translational evaluation [70,71,91].

In line with established guidance, VKAs carry principal risks of bleeding, especially intracranial and gastrointestinal, along with teratogenicity and extensive interaction liability, which together mandate patient education and careful International Normalized Ratio (INR)-based management, potentially augmented by genotype-guided dosing when available [73,74,75,76,77,79]. In contrast, aminocoumarin antibiotics are now predominantly research tools whose safety is managed within laboratory frameworks because toxicity and resistance limit clinical applicability [82,86]. For natural coumarins such as osthol and auraptene, there are promising preclinical findings with limited human pharmacokinetic and exposure–response information, indicating that rigorous dose-ranging, safety, and interaction studies are prerequisites before therapeutic use can be considered [69,70,71,88,91].

In summary, coumarins exhibit a broad range of applications, ranging from clinically indispensable anticoagulants to mechanistic probes of bacterial topoisomerases and promising natural products with multi-target pharmacology. VKAs remain essential yet complex therapies that require individualized, genotype-aware dosing and vigilant monitoring to balance efficacy against bleeding risk, as reflected in public health prioritization [73,74,75,76,77,79,81]. In parallel, aminocoumarins continue to illuminate type II topoisomerase biology despite limited clinical roles, whereas emerging agents such as osthol and auraptene display anti-inflammatory and anticancer activities, including modulation of cancer stemness and drug resistance, that justify further translational research to define clinical viability [69,70,71,82,83,84].

#### Structure-Activity Relationship of Coumarin Substituents

The pharmacological behavior of coumarins can be rationalized through a small set of substituent-dependent rules that relate the biotransformation chemistry described in Section 2 to the biological outcomes summarized above. Hydroxylation is the most consistent activity-enhancing modification: free phenolic hydroxyl groups at the C-6 and C-7 positions increase polarity and confer radical-scavenging/antioxidant capacity, as exemplified by esculetin and umbelliferone, while ortho-dihydroxy (catechol-type) or ortho-hydroxy-methoxy substitution patterns on the aromatic ring markedly potentiate antiproliferative activity relative to mono-substituted analogues, with hydroxylated 3-phenylcoumarins bearing such motifs showing several-fold greater potency than resveratrol against leukemia and lung carcinoma cell lines [92]. Simple hydroxycoumarins, including 6-hydroxycoumarin and 6-nitro-7-hydroxycoumarin, likewise display cytotoxic and cytostatic activity across renal, gastric, and hepatoma-derived cell lines, acting through cyclin-dependent kinase and stress-activated protein kinase pathways. In contrast, methoxylation reduces polarity and increases lipophilicity relative to the corresponding phenol; this can enhance membrane permeability and target engagement, and several structure–activity relationship (SAR) studies report that electron-donating methoxy substituents on coumarin hybrids increase cytotoxic potency by strengthening interactions with the target binding pocket [93,94]. Within the furanocoumarin subclass specifically, anticancer activity has been correlated with methoxy or isoprenyl substitution at C-5 or C-8, as seen in bergapten (5-methoxypsoralen) and xanthotoxin [95].

The furan ring itself introduces a distinct structural rule governed by fusion geometry rather than simple substitution. Linear furanocoumarins (psoralen-type, furan fused at C6–C7) adopt a planar configuration capable of intercalating and photo-crosslinking DNA at pyrimidine bases upon UV exposure, which underlies both their established phototherapeutic use in psoriasis and vitiligo and their inherent phototoxicity liability, whereas angular furanocoumarins (angelicin-type, furan fused at C7–C8) are sterically prevented from bis-intercalation and are consequently explored as lower-toxicity analogues [96]. Detoxification strategies for linear furanocoumarins have exploited this geometric rule directly, using angular isomers, substituents that block the photoreactive α-pyrone double bond, or additional ring annelation to suppress DNA crosslinking while retaining bioactivity [97]. Independently of phototoxicity, the furan-fused scaffold has also been mechanistically linked to anti-inflammatory activity through upregulation of antioxidant enzymes (catalase, superoxide dismutase, glutathione peroxidase) and suppression of iNOS/COX-2 expression [98,99].

Glycosylation, whether O-, C-, or S-linked, follows a consistent solubility–activity trade-off rather than a straightforward activity-enhancing rule: conjugation of a sugar moiety reliably improves aqueous solubility, membrane transport, and chemical or metabolic stability of the coumarin aglycone but frequently attenuates its direct pharmacological potency unless the sugar unit itself participates in target recognition [98,99]. This pattern is undergone by C-glycosylated flavanone analogues, in which glycosylation reduced antimicrobial potency while improving aqueous solubility and predicted pharmacokinetic behavior [100], and in S-glycosylated coumarins (Section 2.4), which enhanced hydrolytic stability and fluorescence suitable for bioimaging while showing negligible cytotoxicity relative to their native counterparts [62]. Taken together, these four substituent classes define a coherent, mechanism-based rule set for coumarin optimization: hydroxylation and *ortho*-dihydroxylation for redox-mediated antiproliferative potentiation, methoxylation for lipophilicity-driven target engagement, furan-ring fusion geometry for tuning phototoxicity and DNA-intercalation capacity, and glycosylation for solubility and bioavailability enhancement at some cost to intrinsic potency. This substituent-activity framework provides a predictive complement to the microbial biotransformation routes discussed in Section 2, since the same reactions catalogued there (hydroxylation, methylation/demethylation, and glycosylation) are precisely the transformations that govern the pharmacological rules outlined here.

### 3.2. Biotransformation in Mechanistic Studies and the Pharmaceutical Potential of Coumarins

**Figure 22:**
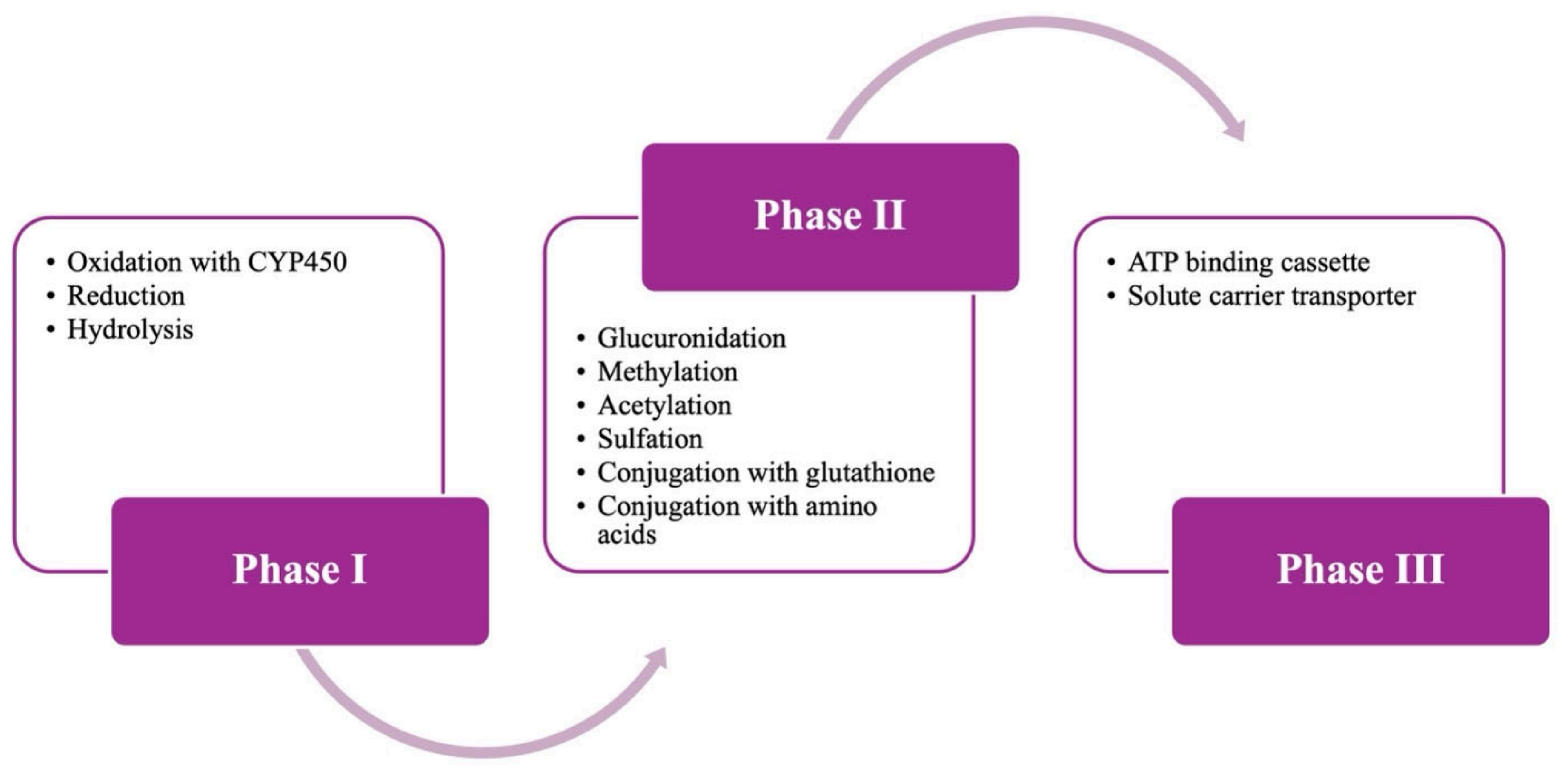
The main phases of xenobiotics metabolism (adapted from Phang-Lyn et al., 2020 [102]).

Biotransformation employs whole or immobilized microbial cells, cell extracts, or isolated enzymes to precisely modify the xenobiotics, such as coumarins. In the context of drug discovery, these microbial systems are invaluable not only for route scouting but also to produce potential metabolites required for pharmacology and safety studies [101,102,103]. Compared to classical chemical synthesis, microbial transformation offers regioselectivity and stereoselectivity, high catalytic efficiency, and reduced catalyst waste. These features are particularly invaluable for late-stage functionalization and the streamlined generation of metabolites [103,104,105,106]. Xenobiotic metabolism generally increases molecular polarity to facilitate excretion, a process containing of three sequential phases as shown in Figure 22.

Phase I introduces or unmasks polar functional groups through oxidation, reduction, or hydrolysis. The major catalysts include cytochromes P450, flavin-containing monooxygenases, esterases, and amidases. Importantly, Phase I may either attenuate biological activity or generate metabolites with equal or greater activity than the parent compound. Furthermore, phase II typically involves conjugation reactions (e.g., glucuronidation, sulfation), which generally reduce pharmacological activity; however, in certain cases, prodrug activation or the formation of reactive, potentially toxic conjugates may also occur. Subsequently, phase III encompasses cellular efflux and excretory processes that remove metabolites from the organism. All these stages determine the exposure and response relationship and safety margins of xenobiotics, including coumarins, across preclinical and clinical settings. From an industrial perspective, any development effort that leverages bioactive coumarins must be preceded by rigorous metabolism studies, since biotransformation underpins pharmacokinetics, pharmacodynamics, and safety profiling [101,102,103].

Conventional metabolism investigations employ in vitro systems such as liver microsomes and hepatocytes, along with in vivo animal models; however, the low abundance of metabolites in complex biological matrices can impede both detection and isolation. In this case, preparative-scale microbial biotransformation, particularly that using filamentous fungi, provides a practical route to obtain milligram-to-gram quantities of authentic metabolites, often with high chemo-, regio-, and stereo-control at comparatively low cost [103,104,105,106]. Owing to their diverse cytochrome P450 repertoires and accessory enzymes, fungi can carry out oxidation, reduction, epoxidation, hydrogenation, hydrolysis, amination, acylation, glycosylation, hydration, methylation, and hydroxylation that partially emulate mammalian metabolic pathways and generate mammal-like metabolites of coumarins [104,105,106].

It is instructive to place microbial biotransformation in direct comparison with conventional chemical synthesis of coumarin derivatives. Chemical routes including Pechmann, Knoevenagel, Perkin, and Kostanecki–Robinson condensations, as well as their modern green-chemistry variants (microwave- and ultrasound-assisted synthesis, solvent-free and mechanochemical methods, and ionic-liquid or deep-eutectic-solvent catalysis), offer high reproducibility, scalability, and established process control, and green modifications of these methods have been shown to improve yield, purity, and energy efficiency relative to classical thermal condensation. However, these chemical methods are generally poorly suited to introducing regio- and stereospecific modifications, particularly hydroxylation at inactivated positions, enantioselective reduction, or site-selective glycosylation, and often require harsh catalysts, elevated temperatures, or multistep protection/deprotection sequences to achieve the selectivity that microbial systems provide directly. Conversely, microbial biotransformation offers regioselectivity, stereoselectivity, and mild reaction conditions unattainable by chemical synthesis (Section 2.1, Section 2.2, Section 2.3 and Section 2.4), but currently lags chemical methods in scalability, batch-to-batch reproducibility, and volumetric productivity. A rational development strategy, therefore, favors a hybrid approach, which is chemical synthesis for constructing or diversifying the core scaffold at scale, followed by microbial biotransformation for late-stage, position-specific functionalization (hydroxylation, demethylation, or glycosylation) that would otherwise require costly protecting-group chemistry [107,108].

In line with established principles of translational research, studies on coumarins and their metabolites should move from structural elucidation and bioactivity screening to incorporate a holistic ADMET (absorption, distribution, metabolism, excretion, and toxicity) assessment supported by both experimental and computational methods. This extends the academic interest; approximately 90% of pipeline failures have been attributed to suboptimal pharmacokinetic properties, thereby underscoring the essential of early ADMET derisking strategies. In recent years, in silico platforms have gained prominence for ADMET prediction because they reduce costs and timelines, prioritize compounds before animal testing, and align with ethical imperatives to minimize animal use [109,110]. Within ADMET, lipophilicity is a key determinant of membrane permeability, plasma protein binding, target engagement, and off-target interactions; accordingly, many studies have explored structure-lipophilicity-activity relationships as a rational basis for optimization [82,111,112,113,114]. These relationships are particularly relevant for coumarins, whose substitution patterns modulate logP/logD, passive diffusion, and metabolic soft spots, thereby shaping both efficacy and safety windows [115,116].

In pharmaceutical formulations, coumarins are typically investigated as active research ingredients rather than used as routinely employed excipients in finished medicinal products. Plant-derived coumarins require purification, characterization, and standardization prior to use in medicines or cosmetics, with quality and safety controls meeting pharmacopeial and regulatory expectations. Safety considerations merit special emphasis at elevated exposures or in susceptible populations; certain coumarins and their derivatives can exhibit phototoxic or hepatotoxic liabilities; thus, systematic risk assessment, including photo safety and drug-induced liver injury (DILI), is crucial prior to clinical translation [102,103].

In summary, integrating microbial biotransformation with modern analytical methods and in silico ADMET tools provides a coherent framework to elucidate coumarin metabolism, generate authentic metabolites, guide structure optimization, and anticipate safety risks. This integration is likely to accelerate the progression of promising coumarin scaffolds from mechanistic enquiry to therapeutic evaluation while improving translational fidelity.

## 4. Limitations in Microbial Transformation

Although the microbial transformation process has significant advantages in various applications, some limitations should be included, such as nutrient limitations and adaptation lag, toxicity of substrates and products, environmental and operational conditions, and challenges in modification systems.

As microbial transformation relies on the ability of microorganisms, the efficacy of the microbial transformation process is influenced by the availability of essential nutrients. Several studies have shown that limitations in important nutrients, including nitrogen and phosphorus, lead to prolonged adaptation lag periods. This prolonged period can significantly reduce the efficiency of microbes in converting certain metabolite compounds [87]. For instance, *Pseudomonas putida* is utilized in the microbial transformation process to hydroxylate coumarins into umbelliferon. In this bioconversion process, the decreasing of nutrient, especially nitrogen, has been shown to prolong the adaptation phase and lead to the delay of the biotransformation reaction. Insufficient nitrogen causes the growth of microbes become slows and reduces the production of necessary enzymes, leading to the decreasing of transformation efficiency [88]. To address this limitation, fed batch or intermittent nutrient feeding can be used to regulate nitrogen and phosphorus to reach the limiting threshold throughout the microbial transformation process, thereby shortening the adaptation lag. Utilizing this fed-batch mixed substrate feeds has been shown to increase biomass and target enzyme activity compared with conventional batch approaches. In addition, this approach has shown predictable scalability from microbioreactor to stirred-tank bioreactor systems [117].

Moreover, the challenge in the microbial transformation of coumarins is the cytotoxicity that leads to the inhibition of microbial growth and metabolic activity. For example, research showed that coumarin concentrations higher than 0.5 g/L can decrease microbial transformation products. Therefore, the selection or engineering of microbial strains is very crucial to overcome this challenge. The higher tolerance of microorganisms will impact the effectiveness of the biotransformation process [118]. Additionally, another coumarin derivates have discovered to selectively inhibit the activity of certain cell lines. For instance, terpene coumarins, which were obtained from microbial transformation by *Mucor polymorphosporus*, showed activity against human gastric cancer cells. While this characteristic is essential for therapeutic applications, it also presents challenges due to the cytotoxic risks involved in microbial transformation processes [46]. Considering these toxicity issues, rather than depending on strain selection, toxicity thresholds can be engineered too. For instance, by employing adaptive laboratory approaches to combine mutagenesis with biosensor-assisted high-throughput screening, can monitor the strains which enhanced the tolerance and biosynthetic capacity simultaneously. Moreover, these adaptive laboratory mutagenesis strategies are an effective way to improve robustness to toxic substrates. Furthermore, in situ product removal or substrate encapsulation also provides other alternatives for process engineering; for example, encapsulating a co-strain with a hydrogel matrix to protect it from the toxic effects of substrate led to increased performance; this strategy can be transferred to coumarin-tolerant strain pairings [119].

The environmental conditions, such as temperature, pH, oxygen, and nutrient availability, substrate or inducer concentrations, significantly affected the microbial transformation processes. Temperature is one of the crucial factors that directly impacts the enzymatic activity of microbial strains. Each microorganism has its optimal temperature range for growth and undergoes biotransformation. The temperature outside of this range will lead to a decrease in microbial activity and might inhibit biotransformation. Higher temperatures can sometimes cause the denaturation of essential enzymes involved, while lower temperatures can reduce microbial metabolic activity [120]. Another factor that has a significant impact on microbial transformation is oxygen availability, as most of the microbial transformation processes are aerobic and the formation of involved enzymes is also dependent on the oxygen content. Monooxygenases and dioxygenases produced by fungi introduce the oxygen atoms into the organic compounds to facilitate the cleavage of aromatic rings and enable the modification of coumarins. These enzymes are known for their high regioselectivity and stereoselectivity, making them essential biocatalysts [121]. Lack of oxygen content can lead to reduced growth rates of microorganisms and disrupt the biotransformation of coumarins [122]. Additionally, nutrient content, such as carbon, nitrogen, potassium, and magnesium, plays a vital role in facilitating microbial growth and optimizing the microbial transformation process. Each microorganism has its specific nutrient requirement for its growth and metabolic activity. Imbalanced nutrient content can inhibit microbial activity and microbial transformation processes, leading to decreased production of metabolite compounds [123]. High substrate concentrations can be toxic to microorganisms or inhibit the growth of microorganisms, leading to decreased efficiency of biotransformation. Optimizing the concentration of substrate that is added to the inoculum is crucial for an effective microbial transformation process. Mitigating the side effects of high substrate concentrations by controlling the levels of substrate below inhibitory thresholds can be done using fed-batch fermentation. To address the limitations regarding the environmental conditions, systematic optimization approaches such as design of experiments and response surface methodology testing [124].

In addition, the co-culture and cell-factory methods discussed in Section 2.3 also address the scale-up process, since the modified pathways for coumarins have already been applied in host strains such as *E. coli* and *S. cerevisiae*, providing scalable and environmentally friendly processes [57]. In summary, process engineering, including fed-batch nutrient control, in situ product removal, and encapsulation with engineered strains or enzymes, provide a promising approach to address the limitations of biotransformation, particularly on an industrial scale.

## 5. Conclusions

Despite all the progress in microbial transformation, several issues remain unresolved. First, the enzymes involved in many of the microbial transformations, such as HcdA, HcdB, HcdC, and SaOMT2, have been characterized only in a small number of strains; their regioselectivity trait also remain mostly unmapped. Second, most research about microbial transformation only used single strains and laboratory-scale processes; standardized data on yield, regioselectivity, and robustness through different microbial systems are lacking, leading to difficulty in selecting biocatalysts for industrial development. Third, there are only a few early stages of research for engineering coumarin-specific enzymes and coumarin de novo biosynthesis. Lastly, few studies connect the microbial transformation products described in this review to structure–activity evaluation, so the pharmacological consequences of many of these biotransformation reactions remain uncharacterized. Addressing these gaps through targeted enzyme discovery, cross-strain benchmarking, protein/pathway engineering, and integration with pharmacological profiling represents the most promising direction for translating microbial coumarin biotransformation from a descriptive research area into a predictable industrial biocatalysis platform.

Crucially, the bottlenecks detailed in Section 4 are actively being resolved. Synthetic biology techniques, including engineered microbial consortia and single-host pathway reconstruction, overcome the scale-up and contamination issues of sequential fermentation. Meanwhile, enzyme and strain engineering, via site-directed mutagenesis and adaptive laboratory evolution, directly target low specificity and cytotoxicity, while in situ product removal prevents feedback inhibition. Implementing these integrated approaches is successfully driving coumarin biotransformation away from isolated, strain-specific research toward a predictable, industrially viable biocatalytic platform.

In conclusion, microbial transformation provides a significant and efficient approach to modify bioactive compounds such as coumarins through specialized microbial enzymatic mechanisms. This method serves as an efficient, versatile, and environmentally friendly biocatalytic reaction to execute complex chemical transformations that are often difficult to mimic through traditional chemical synthesis. This biotransformation primarily involves various enzymatic reactions, as summarized in Table 1 below:

More broadly, the microbial transformation reactions discussed in this review, including lactone reduction, hydroxylation, methylation/demethylation, and glycosylation, are not only mechanistically informative but are directly applicable to the synthesis of valuable end products. For instance, dihydrocoumarins and melilotol-type lactones for the flavor and fragrance industry, hydroxylated and glycosylated coumarins with improved solubility and bioactivity for pharmaceutical development, and stereo-defined metabolites for use as authentic reference standards in drug metabolism studies. Positioning microbial biotransformation as a complementary, late-stage functionalization tool alongside conventional chemical synthesis, rather than as a competing approach, offers the most alternative ways to translate the reactions described in this review into scalable production of valuable coumarin-derived products.

## Figures and Tables

**Table 1:** Summary of reactions in microbial transformation of coumarins.

No.	Reaction Mechanism	Microorganism Involved
1	Lactone ring reduction and downstream degradation	*Aspergillus* spp., *Aspergillus* sp. PTCC 5266, *Cunninghamela elegans*, *Glomerella cingulata* NBRC 5952, *Penicillium camemberti*, *Kluyveromyces marxianus*, *Torulaspora delbrueckii*.
2	Hydroxylation	*Cunninghamela elegans* NRRL 1392 and ATCC 36112, *Aspergillus* spp., *Mucor spinous*, *Alternaria longipes*, *Mucor polymorphosporu*.
3	Methylation and demethylation	*Streptomyces avertimilis*, *Streptomyces griseus*, *Candida tropicalis*, *Candida albicans*
4	Glycosylation	*Bacillus licheniformis*, *Angelica decursiva, Streptomyces* spp., *Pseudomonas putida*, *Thermomyces lanuginosos*

## Data Availability

No new data were created or analyzed in this study. Data sharing is not applicable to this article.
